# Variance component analysis of circulating miR-122 in serum from healthy human volunteers

**DOI:** 10.1371/journal.pone.0220406

**Published:** 2019-07-26

**Authors:** Jennifer Vogt, Daniel Sheinson, Paula Katavolos, Hiroko Irimagawa, Min Tseng, Kathila R. Alatsis, William R. Proctor

**Affiliations:** 1 Department of Safety Assessment, Genentech, Inc., South San Francisco, California, United States of America; 2 Nonclinical Biostatistics, Genentech, Inc., South San Francisco, California, United States of America; Institut de Pharmacologie Moleculaire et Cellulaire, FRANCE

## Abstract

Micro-RNA (miR)-122 is a promising exploratory biomarker for detecting liver injury in preclinical and clinical studies. Elevations in serum or plasma have been associated with viral and autoimmune hepatitis, non-alcoholic steatohepatitis (NASH), hepatocellular carcinoma, and drug-induced liver injury (DILI). However, these associations were primarily based upon population differences between the disease state and the controls. Thus, little is known about the variability and subsequent variance components of circulating miR-122 in healthy humans, which has implications for the practical use of the biomarker clinically. To address this, we set out to perform variance components analysis of miR-122 in a cohort of 40 healthy volunteers. Employing a quantitative real-time polymerase chain reaction (qRT-PCR) assay to detect miR-122 and other circulating miRNAs in human serum, the relative expression of miR-122 was determined using two different normalization approaches: to the mean expression of a panel of several endogenous miRNAs identified using an adaptive algorithm (miRA-Norm) and to the expression of an exogenous miRNA control (*Caenorhabditis elegans* miR-39). Results from a longitudinal study in healthy volunteers (N = 40) demonstrated high variability with 117- and 111-fold 95% confidence reference interval, respectively. This high variability of miR-122 in serum appeared to be due in part to ethnicity, as 95% confidence reference intervals were approximately three-fold lower in volunteers that identified as Caucasian relative to those that identified as Non-Caucasian. Variance analysis revealed equivalent contributions of intra- and inter-donor variability to miR-122. Surprisingly, miR-122 exhibited the highest variability compared to other 36 abundant miRNAs in circulation; the next variable miRNA, miR-133a, demonstrated a 45- to 62-fold reference interval depending on normalization approaches. In contrast, alanine aminotransferase (ALT) activity levels in this population exhibited a 5-fold total variance, with 80% of this variance due to inter-donor sources. In conclusion, miR-122 demonstrated higher than expected variability in serum from healthy volunteers, which has implications for its potential utility as a prospective biomarker of liver damage or injury.

## Introduction

Twenty-five years ago, Ambros and colleagues discovered and characterized the first microRNA [1]. Since then, these small noncoding 22-nucleotide miRNAs have been shown to be important mediators in epigenetic, transcriptional, and post-transcriptional gene regulation [2, 3], as well as cellular proliferation, differentiation, metabolism, infectious and non-infectious disease progression, apoptosis and metastasis [2, 4–6]. In addition, miRNAs are known to be highly conserved across several species [4, 7–9] and are relatively stable in serum and plasma [10]. Accordingly, circulating miRNAs are actively being pursued as both therapeutic targets as well as biomarkers of disease and toxicities. In circulation, they exist as two predominant species as either protein bound (in particular, bound to Argonaut-2 [11]), or encapsulated in extracellular vesicles or exosomes [4]. These different circulating species are believed to be leakage markers from necrotic or damaged cells and tissues, or they may originate from active secretion processes [5].

One of the most widely studied miRNAs to date is miR-122. It has been characterized as one of the most abundant miRNAs in any tissue, being present at over 130,000 copies per cell in human hepatocytes [12] and comprising over 70% of the total liver miRNA population [8, 12, 13]. Regulation of miR-122 is tightly controlled by liver-specific transcription factors, including hepatocyte nuclear factor (HNF)-1α, HNF-3β, HNF-4α, and HNF-6 [14–17], which likely drive its liver specificity in regards to tissue expression. Functionally, miR-122 is involved with cholesterol and glucose metabolism [13], circadian rhythm [13, 18, 19], hepatocyte differentiation [14], and lipogenesis [20, 21]. In addition, the sequence of mature miR-122 is conserved across all species in which it has been detected [12–14, 22].

These factors, in addition to its enrichment in the liver, make miR-122 a promising biomarker of hepatocellular injury, hepatic biology, and liver diseases. The utility of miR-122 in this context was first reported by Wang et al. in 2009 [23], where they demonstrated that circulating miR-122 is elevated in the plasma of mice following acute acetaminophen-induced liver injury. Interestingly, these elevations preceded significant increases in circulating transaminase levels. Over the past 10 years, alterations of miR-122 levels in circulation have been observed in patients with drug-induced liver injury (DILI) [24–27], non-alcoholic steatohepatitis (NASH) [20, 28] non-alcoholic fatty liver disease (NAFLD) [29], hepatocellular carcinoma [30, 31], autoimmune hepatitis [32], and viral hepatitis [33–35]. Additionally, recent work has suggested that exosome encapsulated miR-122 may be an early secreted biomarker in liver disease and injury, where elevated circulating exosome encapsulated miR-122 that preceded transaminase increases in mice following acute acetaminophen treatment [23]. This observation supported a potential role of miR-122 as an active signaling molecule during early injury. Similarly, the exosome encapsulated miR-122 has been postulated to be a useful biomarker for differentiating liver impairments and pathologies with potential prognostic benefits in relation to protein bound species, in conjunction with more traditional biomarkers [36–38]. Taken together, circulating miR-122 in regards to total levels, protein bound, or exosome fraction, appears to have the potential to be a useful biomarker for detecting liver injury pre-clinically and in a clinical setting.

Although miR-122 has demonstrated specificity, sensitivity, and translatability as a biomarker of liver injury or hepatic impairment [24, 39, 40], there are few studies that specifically address the variability and population reference intervals of circulating miR-122 that are integral for use as a prognostic biomarker in the clinic. In particular, there are few reports that address intra- and inter-individual fluctuations of circulating miR-122 levels over time, or establish upper limits of normal (ULN) for this marker in a clinical setting. While studies have demonstrated significant elevations in disease-state patients of circulating miR-122 levels, comparisons often are performed on population means in comparison to healthy volunteers or non-liver disease patients [26, 27, 29, 41]. However, a recent publication by Church et al. (2019) investigated the performance of miR-122 against several other known exploratory biomarkers of liver injury [25]. A surprising finding in this study was high inter- and intra-subject variability of miR-122 from two healthy volunteer cohorts, where the 95% confidence intervals spanned 38- to 78-fold ranges in absolute miR-122 levels from both cohorts, respectively [25]. The investigators concluded that the large inter- and intra-subject variation in miR-122 may complicate its interpretation in the clinic, while acknowledging that this biomarker is likely still valuable in certain contexts of use such as in the setting of acetaminophen-induced hepatotoxicity [24, 25]. However, it remains unclear whether the method used to quantify miR-122 in circulation affects this conclusion, as there are currently no standard or universally accepted practices for quantifying or normalization of microRNA data [42–44]. Moreover, it is not known if the high variability of circulating miR-122 observed in humans is consistent with other circulating miRNAs in those patients, as only miR-122 was assessed in this context.

In the following report, we evaluated circulating levels of miR-122 as well as more than 30 other highly abundant circulating miRNAs in healthy volunteers to determine overall intra- and inter-subject variability. To accomplish this, we measured circulating miRNAs in human serum via a quantitative real-time polymerase chain reaction (qRT-PCR)-based assay using two different normalization approaches. We determined relative expression of circulating miR-122 normalized to the mean expression of a panel of endogenous miRNAs selected retrospectively using an unbiased adaptive algorithm, as well as normalized to a traditionally employed exogenous miRNA spike-in *Caenorhabditis elegans* miR-39 (*C eleg* miR-39) control. The results and conclusions presented here demonstrate that miR-122 levels in serum from healthy volunteers exhibited high intra- and inter-subject variability regardless of normalization approaches. The high degree of variance we observed appeared to be specific to miR-122, as the total variance was greater than all other highly abundant circulating miRNAs profiled, or that of circulating liver transaminases alanine transaminase (ALT) and aspartate transaminase (AST). This report, in concordance with recently published work [25], supports that high inter- and intra-subject variance of circulating miR-122 may limit the clinical utility of this exploratory biomarker for prospective monitoring of liver injury.

## Materials and methods

### Serum samples and processing

Whole blood samples were collected from ostensibly healthy, fasted Genentech donors enrolled in the company’s Employee Donation Program. The study followed 42 individuals (19 females, 23 males, ranging from 29 to 62 years of age) for a series of 6 blood draws, to be collected in the mornings between 7 and 10am, spaced one to two weeks apart. Exclusion criteria included alcohol or acetaminophen use within the previous 24 hours. Informed consent was obtained from all individual participants included in the study. The human specimens (e.g. blood products such as serum) used for subsequent biomarker analysis were obtained from volunteers under the Genentech Employee Donation Program (“Samples for Science”), under approval from the Western Institutional Review Board. Demographic information (age, gender, and ethnicity) for these participants was collected. Whole blood was collected in 8 mL Vacutainer serum separation tubes (Becton-Dickinson, Franklin Lakes, NJ) in the absence of any anticoagulants, and centrifuged at 4°C for 10 minutes at 2000 x g. Serum was processed the morning of sample collection, aliquoted, transferred into RNase free tubes, and immediately frozen on dry ice before storage at -80°C for subsequent RNA extraction, isolation and analysis. Frozen aliquots were stored at -80°C for up to six months prior to miRNA extraction with no additional freeze-thaw cycles. The remainder of serum was stored at -80°C for follow-up use if needed. An aliquot of each serum sample was submitted to Quest Diagnostics (West Hills, CA) and a standard clinical chemistry panel was performed on each sample using a Beckman Coulter AU 5800 or AU 4800 analyzer (Beckman Coulter Inc., Brea, CA, USA) using Beckman Coulter assay reagents. The Beckman enzymatic assays used to measure ALT and AST activities did not use Pyridoxal phosphate (P-5-P) co-factor in assessment of catalytic activity of these transaminases [45].

### RNA extraction and qRT-PCR analysis

#### miRNA isolation

All miRNA isolations were performed according to the following protocol, using miRNeasy Serum/Plasma Kit (Qiagen, Valencia, CA) and automation on QiaCube (Qiagen) following manufacturer’s instructions. Briefly, serum samples (100μL) were thawed on ice and lysed using 500μL QIAzol Lysis reagent. Following lysis, 5.6x10^8^ copies of exogenous *C eleg* miR-39 control were spiked into each sample to serve as a quality control for extraction efficiency. 100μL of chloroform was added per sample for RNA binding and phase separation. Following centrifugation, 350μL of RNA-containing aqueous phase was removed to fresh tubes and transferred to QiaCube for automated miRNA isolations. Freshly isolated miRNA-containing eluate (15μL) was immediately frozen on dry ice and stored at -80°C for subsequent qRT-PCR analysis.

#### miRNA reverse transcription

The expression of miR-122, exogenous *C eleg* miR-39, and a panel of 80 other miRNAs were assessed using 96x96 Dynamic Array Integrated Fluidics Chips (Fluidigm Corporation, South San Francisco, CA) for qRT-PCR analysis. The panel of miRNAs evaluated and their corresponding primer sequences are represented in Table 1. Reverse transcription (RT) was accomplished using TaqMan miRNA Reverse Transcription Kit (Thermo Fisher, Waltham, MA). RT reactions were set up on ice in 96 well skirted PCR plates (Thermo Fisher), following published protocols [46]. 2μl of miRNA were added to the following 5.7μL volume of RT Master Mix: 0.1μL 100mM dNTPs containing dTTP, 0.1μL (2U) RNase Inhibitor, 0.75μL 10x Reverse Transcription Buffer, 0.75μL (37.5U) MultiScribe Reverse Transcriptase, and 4μL of targeted, multiplexed reverse primers (pooled to allow final concentration of each primer at 0.05x). Incubation for cDNA conversion was 16°C for 30 minutes, 42°C for 30 minutes, 85°C for 5 minutes. After cooling to 4°C, the freshly transcribed cDNA was stored at -20°C until further processing and analysis could occur.

**Table 1:** Panel of miRNAs and their corresponding primer/probe sequences.

miRNA name	Probe Sequence	miRNA name	Probe Sequence	miRNA name	Probe Sequence
Cel-miR-39	UCACCGGGUGUAAAUCAGCUUG	miR-181a	AACAUUCAACGCUGUCGGUGAGU	miR-29c-3p	UAGCACCAUUUGAAAUCAGUGUU
miR-122	UGGAGUGUGACAAUGGUGUUUG	miR-181c	AACAUUCAACCUGUCGGUGAGU	miR-301	UAGCACCAUUUGAAAUCGGUUA
miR-let7b	UAUUGCACUUGUCCCGGCCUGU	miR-182	UUUGGCAAUGGUAGAACUCACACU	miR-301a	GCUCUGACUUUAUUGCACUACU
miR-17-5p	CAAAGUGCUUACAGUGCAGGUAG	miR-187-3p	UCGUGUCUUGUGUUGCAGCCGG	miR-30c	UGUAAACAUCCUACACUCUCAGC
miR-19b-3p	UGUGCAAAUCCAUGCAAAACUGA	miR-187-5p	GGCUACAACACAGGACCCGGGC	miR-326	CCUCUGGGCCCUUCCUCCAG
miR-20a-5p	UAAAGUGCUUAUAGUGCAGGUAG	miR-18a-5p	UAAGGUGCAUCUAGUGCAGAUAG	miR-335-5p	UCAAGAGCAAUAACGAAAAAUGU
miR-20b	CAAAGUGCUCAUAGUGCAGGUAG	miR-191	AACAUUCAACGCUGUCGGUGAGU	miR-339-3p	UGAGCGCCUCGACGACAGAGCCG
miR-100	AACCCGUAGAUCCGAACUUGUG	miR-192	CAACGGAAUCCCAAAAGCAGCUG	miR-339-5p	UCCCUGUCCUCCAGGAGCUCACG
miR-103-3p	AGCAGCAUUGUACAGGGCUAUGA	miR-193a-5p	UGGGUCUUUGCGGGCGAGAUGA	miR-342	UCUCACACAGAAAUCGCACCCGU
miR-106a	AAAAGUGCUUACAGUGCAGGUAG	miR-196b	UAGGUAGUUUCCUGUUGUUGGG	miR-374	UUAUAAUACAACCUGAUAAGUG
miR-106b	UAAAGUGCUGACAGUGCAGAU	miR-200a-3p	UAACACUGUCUGGUAACGAUGU	miR-374b*	CUUAGCAGGUUGUAUUAUCAUU
miR-107	AGCAGCAUUGUACAGGGCUAUCA	miR-206	UAAUACUGCCUGGUAAUGAUGA	miR-375	UUUGUUCGUUCGGCUCGCGUGA
miR-10a	UACCCUGUAGAUCCGAAUUUGUG	miR-21	UAGCUUAUCAGACUGAUGUUGA	miR-378a-3p	ACUGGACUUGGAGUCAGAAGGC
miR-125a-5p	UCCCUGAGACCCUUUAACCUGUGA	miR-210	CUGUGCGUGUGACAGCGGCUGA	miR-378a-5p	CUCCUGACUCCAGGUCCUGUGU
miR-125b-5p	UCCCUGAGACCCUAACUUGUGA	miR-213	ACCAUCGACCGUUGAUUGUACC	miR-424	CAGCAGCAAUUCAUGUUUUGAA
miR-130a	CAUUAUUACUUUUGGUACGCG	miR-221-3p	UUGUGCUUGAUCUAACCAUGU	miR-451	AAACCGUUACCAUUACUGAGUU
miR-133a	UAACAGUCUACAGCCAUGGUCG	miR-222-3p	AGCUACAUCUGGCUACUGGGU	miR-483-5p	AAGACGGGAGGAAAGAAGGGAG
miR-135a	UAUGGCUUUUUAUUCCUAUGUGA	miR-223	AGCUACAUUGUCUGCUGGGUUUC	miR-484	UCAGGCUCAGUCCCCUCCCGAU
miR-139-5p	UCUACAGUGCACGUGUCUCCAG	miR-22-3p	AAGCUGCCAGUUGAAGAACUGU	miR-500a-5p	UAAUCCUUGCUACCUGGGUGAGA
miR-143	UUUGGUCCCCUUCAACCAGCUG	miR-23a-3p	UGUCAGUUUGUCAAAUACCCCA	miR-511-3p	AAUGUGUAGCAAAAGACAGA
miR-145	UACAGUAUAGAUGAUGUACU	miR-24	AUCACAUUGCCAGGGAUUACC	miR-511-5p	GUGUCUUUUGCUCUGCAGUCA
miR-146a-5p	UGAGAACUGAAUUCCAUGGGUU	miR-26b	UUCAAGUAAUUCAGGAUAGGU	miR-629-5p	UGGGUUUACGUUGGGAGAACU
miR-146b-5p	UGAGAACUGAAUUCCAUAGGCU	miR-27a-3p	AGCAGAGGCAGAGAGGCUCAGG	miR-9	UCUUUGGUUAUCUAGCUGUAUGA
miR-147	GUGUGUGGAAAUGCUUCUGC	miR-27a-5p	AGGGCUUAGCUGCUUGUGAGCA	miR-92a	AUAAAGCUAGAUAACCGAAAGU
miR-147b	GUGUGCGGAAAUGCUUCUGCUA	miR-28-3p	CACUAGAUUGUGAGCUCCUGGA	miR-99a-5p	AACCCGUAGAUCCGAUCUUGUG
miR-150	UGAGAACUGAAUUCCAUGGGUU	miR-28-5p	UUCACAGUGGCUAAGUUCCGC	miR-let-7d	UGAGGUAGUAGGUUGUGUGGUU
miR-155	UUAAUGCUAAUCGUGAUAGGGGU	miR-29a-3p	UAGCACCAUCUGAAAUCGGUUA		
miR-16	UUAAUGCUAAUCGUGAUAGGGGU	miR-29b-3p	UAGCACCAUUUGAAAUCAGUGUU		

#### Pre-PCR amplification

TaqMan miRNA gene expression assays (Thermo Fisher) outlined in Table 1 were pooled so each assay was present at a final concentration of 0.2x. Per reaction, 2.5μL TaqMan PreAmp Master Mix (2x) and 1.25μL of the pooled 0.2x assays were combined with 1.25μL of cDNA, for a total volume of 5μL. Pre-amplification PCR consisted of one cycle each of 95°C for 10 minutes, 2 minutes at 55°C, and 2 minutes of 72°C. This was followed by 15 cycles of 95°C for 15 seconds and template extension at 60°C for 4 minutes. One final 10-minute incubation at 99°C was followed by 4°C hold until the plate was collected. Upon completion, all pre-amplification reactions were diluted 1:5 with DNA Suspension Buffer (Teknova, Hollister, CA), for a final volume of 25μL. Samples were stored at -20°C until subsequent qRT-PCR steps could be completed.

#### qRT-PCR analysis

Each sample was run in quadruplicate against a panel of 82 miRNA assays. 2.75μL from the diluted pre-amplification plate was combined with a qPCR Master Mix containing 2.5μL of 2x TaqMan Universal MasterMix II without Uracil-N glycosylase (Thermo Fisher) and 0.25μL of 20x Gene Expression Loading Reagent (Fluidigm Corporation). Volumes were scaled accordingly to account for replicates and pipetting error. 20x TaqMan assays were prepared for chip loading by combining 3μL each assay with 3μL 2x Assay Load Reagent (Fluidigm Corporation). Each 96x96 Dynamic Array chip was primed with a fixed volume of control line fluid on an HX Integrated Fluidic Circuit controller (Fluidigm Corporation). Following the prime step, 5.2 μL of sample mix was loaded, followed by 5 μL of each TaqMan assay mix per manufacturer’s protocols. The IFC controller was then used to prime the prepared chip, which was then loaded onto a BioMark instrument (Fluidigm Corporation) for quantitative thermal cycling using the 96x96 Standard V2 40-cycle protocol.

### Data normalization and analysis

All expression data was reviewed using Fluidigm Real-Time PCR Analysis software (Fluidigm Corporation). Cycle thresholds (Ct) for each assay were determined using user defined baseline derivative, and samples were failed if the Ct was above 25 or below 4. Only technical replicates with non-missing Ct values that passed initial quality control and were Ct <25 were included in the sample mean calculation. Samples with less than 2 technical replicates meeting these criteria were excluded from the analysis. All calculations were performed in R [47]. Normalized Ct (ΔCt) values were calculated using two different methods: MiRA-norm and *C eleg* miR-39 spike-in. Using the MiRA-norm method, ΔCt values were calculated for each sample by subtracting an assay’s Ct value by the mean Ct among genes selected by applying the MiRA-norm package in R [48]. The MiRA-norm algorithm selects normalizing genes by examining the correlation among genes across samples and minimizing measurement error. Using the spike-in method, ΔCt values were calculated for each sample by subtracting an assay’s Ct value by the Ct value for exogenous *C eleg* miR-39. Reference intervals were calculated based on longitudinal samples collected from 40 volunteers at 6 separate visits (N = 240). For each assay, a random-effects model which assumed normally distributed random effects for each individual donor and normally distributed within-donor deviations was fit to the Ct values using the lme4 package in R [49]. Reference intervals capturing 95% of the data were calculated using the model estimate of the total variance (sum of between- and within- donor variance) and by assuming a t-distribution for the Ct values. The degrees of freedom for the t-distribution were determined by calculating the intra-donor correlation coefficient to derive the effective size of the sample [50, 51]. For all statistical analyses, we only processed gene expression data for any specific miRNA if it was at least 95% complete across the entire sample set.

## Results

### Establishment of reference intervals for miR-122 and other circulating miRNAs

**Fig 1:**
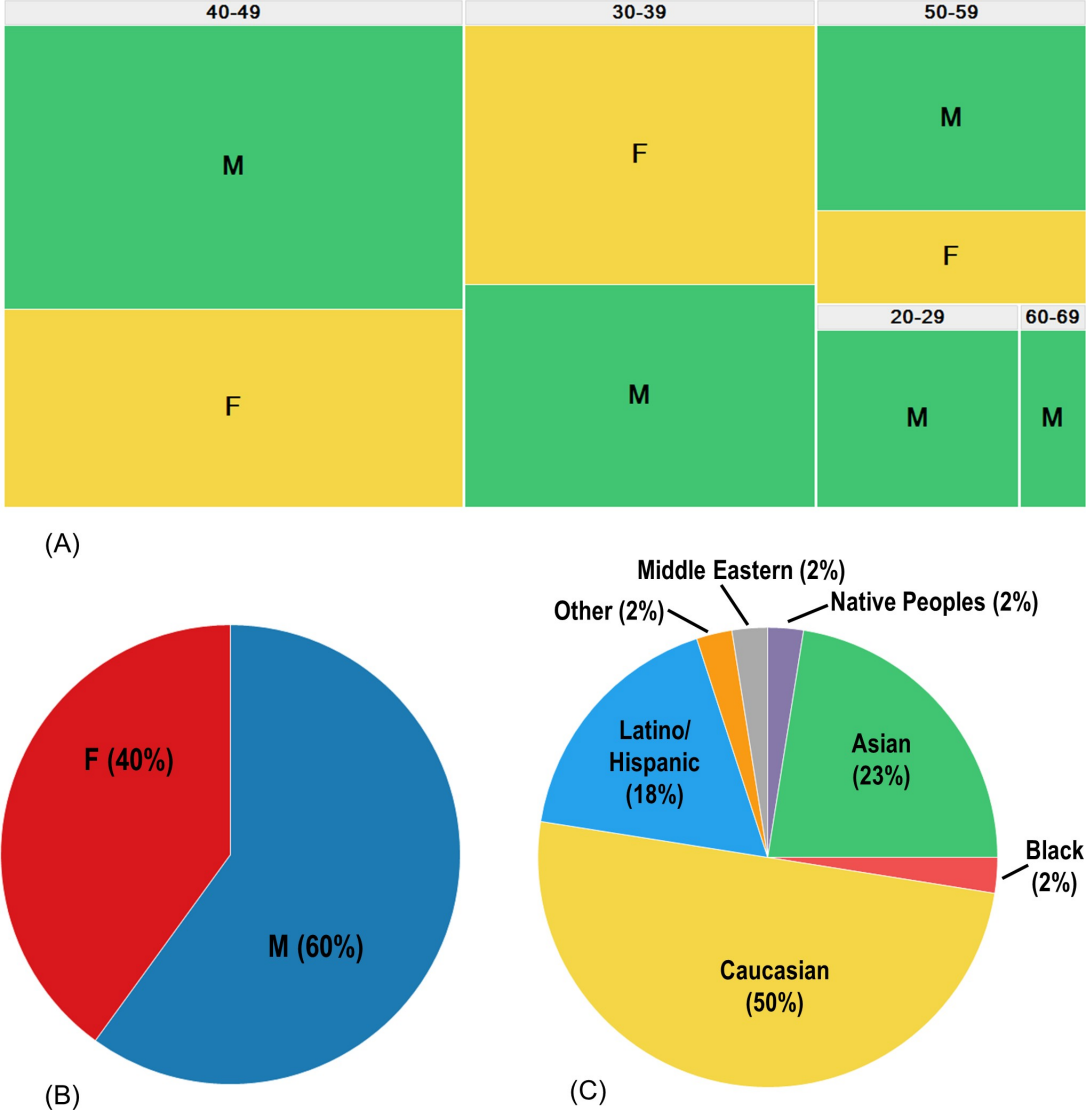
Summary of the demographics of study volunteers. Healthy volunteers (N = 40) were enrolled in this study, donating 6 serum samples over 10-week period or less. (A) Overall age and gender metrics for this cohort, where the area of each rectangle is proportional to the number of volunteers that segregate into each age group. (B) Overall gender split: the cohort consisted of 16 females and 24 Males. (C) Summary of the individually-identified ethnic demographics for the volunteer cohort.

Our study consisted of 42 volunteers from the Genentech Employee Donation Program giving 6 blood donations over six to ten weeks. A summary of the demographics of the volunteers can be found in Fig 1 and times in the morning of each blood collection in S1 Table. Samples were excluded from subsequent analysis based on review of standard clinical chemistry parameters: two subjects were removed from our analysis due to two-fold or greater elevations in either alanine aminotransferase (ALT) activities (Donor 8) compared to the upper limit of normal (ULN) of the testing laboratory’s reported reference intervals, or multiple, atypically elevated blood glucose (all 6 values were above 200mg/dL, Donor 42). All remaining samples (N = 40 volunteers, donating six samples each for N = 240 total samples) were included in this study based on normal clinical chemistry results, including liver transaminase levels (S2 Table). These 240 samples were used to establish 95% reference intervals for the panel of miRNAs (including miR-122) as well as circulating levels of transaminases (ALT and AST). We used the MiRA-norm algorithm to normalize the gene expression of miR-122 and other miRNAs in our panel to the mean expression of a panel of five miRNAs whose expression moved consistently across the sample set [48]. MiRA-norm identified miR-let7b, miR-17-5p, miR-19b-5p, miR-20a-5p, and miR-20b as the most appropriate miRNAs for expression normalization. A depiction of the movement of the expression of miRNAs, including the 5-selected endogenous miRNAs, and the mean values of these are represented in S1 Fig. The selection of these five miRNAs for normalization was consistent over 4 other human serum sample sets from patients in a controlled clinical setting analyzed by identical qRT-PCR methods and the panel of miRNAs outlined here (data not shown). For all comparisons following, the relative expression of miR-122 and other abundant miRNAs profiled will be reported normalized to the mean expression of the 5 miRNAs listed above (referred to as “MiRA-Norm”) as well as to exogenous spike-in miRNA (*C eleg* miR-39).

**Fig 2:**
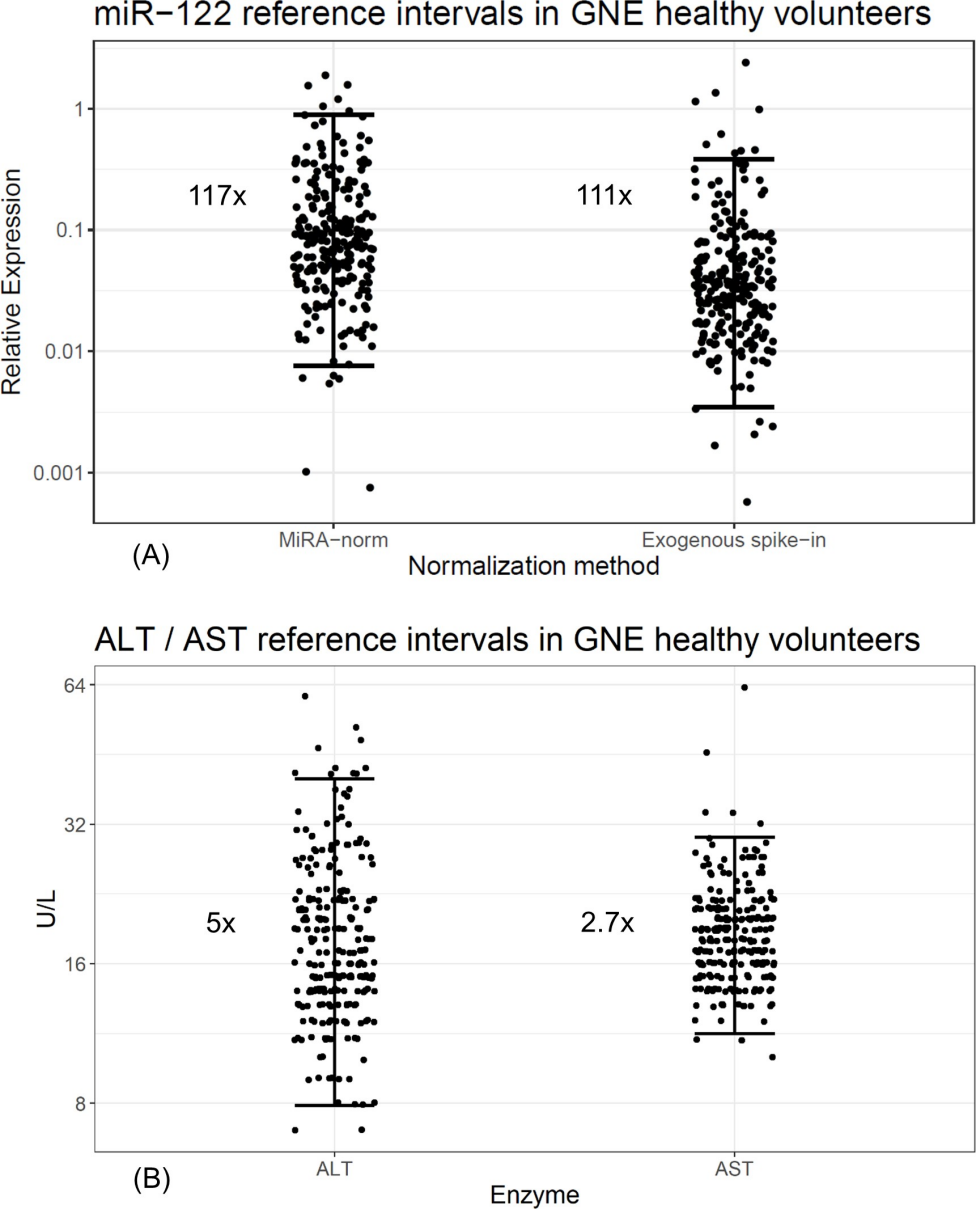
The 95% confidence reference ranges of miR-122, alanine transaminase (ALT), and aspartate transaminase (AST) in serum from healthy volunteers. (A) The relative serum expression of miR-122 normalized to the mean expression of a panel of five endogenous miRNAs (“MiRA-norm”, left) or to the exogenous *C eleg* miR-39 exogenous spike-in (right). Brackets represent the 95% confidence reference ranges for miR-122 serum expression, where miR-122 relative expression spanned 117- and 111-fold when normalizing to miRNA-norm or to *C eleg* miR-39 expression, respectively. (B) Serum expression of ALT (left) and AST (right) and their corresponding reference ranges. The reference range for ALT and AST were determined to be 5.0- and 2.7-fold, respectively.

**Fig 3:**
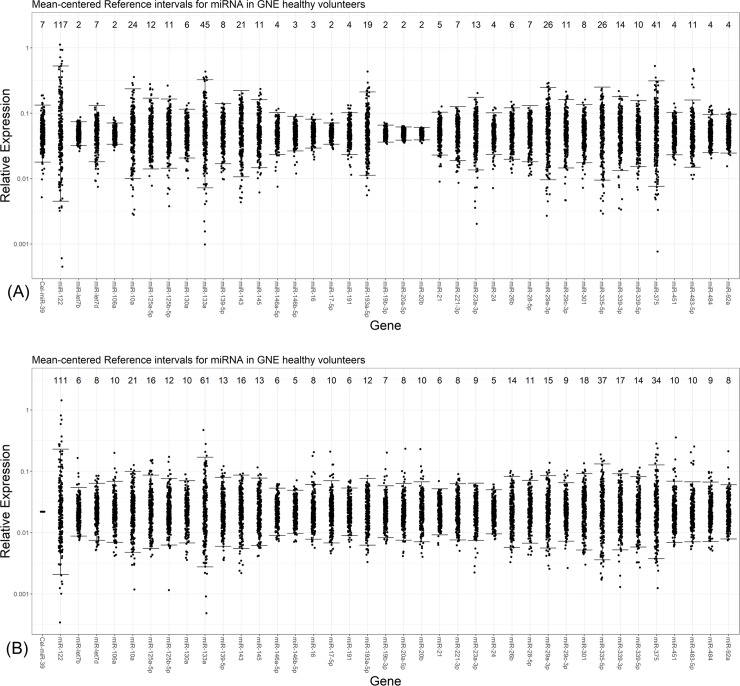
Mean-centered 95% confidence reference intervals of 38 abundant miRNAs relative to miR-122 in serum from healthy volunteers. The relative expression of 38 abundant miRNAs in human serum from healthy volunteers, including miR-122, are depicted as mean centered relative expression for miRNAs normalized using MiRA-norm (A) or exogenous *C eleg* miR-39 (B). The 95% reference intervals are represented in both panels with N = 240 samples per plot. The relative fold-change in the reference range for each 95% confidence reference interval is displayed above each miRNA.

Circulating levels of miR-122 in serum demonstrated high variability (Fig 2A), with reference intervals having 117- and 111-fold differences between lower and upper bounds of relative expression when normalized using MiRA-norm or *C eleg* miR-39 (95% CI), respectively. In contrast, the lower to upper bound fold differences for ALT and AST activities in this sample set were 5.0 and 2.7, respectively (95% CI) (Fig 2B), which is consistent with historical, established ranges in normal populations [52, 53]. We then examined the reference intervals of the other miRNAs in this 240-sample set. Only circulating miRNAs that were accurately measured in at least 95% of the 240 samples were included in further statistical analysis and reference interval calculations. As such, only 36 of 80 miRNAs outlined in Table 1 met the appropriate criteria (refer to S3 Table). The 95% reference intervals for these miRNAs (not including miR-122) varied from a range of approximately 2- to 45-fold when applying the MiRA-norm approach, and approximately 6- to 62-fold when normalized to exogenous *C eleg* miR-39 (Fig 3A and 3B, Table 2). Based on this, miR-122 was the most variable of all the abundant 36 circulating miRNAs in human serum regardless of normalization approaches (Fig 3A and 3B, Table 2). The next most variable miRNA (miR-133a) demonstrated a reference range of 45-fold and 62-fold when normalized to MiRA-norm and *C eleg* miR-39, respectively. Interestingly, the 5 miRNAs (not including miR-122) with the widest 95% reference intervals (miR-133a, miR-29a, miR-375, miR-335-5p, miR-10a) retain this characteristic regardless of the normalization strategy employed (Fig 3A and 3B, Table 2)

**Table 2:** The 95% confidence reference ranges of other 38 abundant miRNAs in relation to miR-122 in serum from healthy volunteers.

Gene	MiRA-norm					Normalized to Cel-miR-39 (spike-in)				
	Mean Rel. Expr.	95% CI Rel. Expr.	GeoMean (dCt)	95% CI (dCt)	Fold-change in Rel. Expr.	Mean Rel. Expr.	95% CI Rel. Expr.	GeoMean (dCt)	95% CI (dCt)	Fold-change in Rel. Expr.
Cel-miR-39	2.248	(0.8229, 6.1411)	-1.17	(-2.62, 0.28)	7.5	1	NA	0	NA	NA
miR-122	0.0822	(0.0076, 0.8891)	3.6	(0.17, 7.04)	116.9	0.0366	(0.0035, 0.3849)	4.77	(1.38, 8.17)	110.7
miR-let7b	0.6149	(0.4057, 0.9322)	0.7	(0.1, 1.3)	2.3	0.2736	(0.11, 0.6804)	1.87	(0.56, 3.18)	6.2
miR-let7d	0.0534	(0.0198, 0.144)	4.23	(2.8, 5.66)	7.3	0.0237	(0.0081, 0.0691)	5.4	(3.85, 6.94)	8.5
miR-106a	4.0539	(2.7854, 5.9)	-2.02	(-2.56, -1.48)	2.1	1.8034	(0.5732, 5.6732)	-0.85	(-2.5, 0.8)	9.9
miR-10a	0.0052	(0.0011, 0.0252)	7.59	(5.31, 9.87)	23.6	0.0023	(5e-04, 0.0105)	8.76	(6.57, 10.96)	21.0
miR-125a-5p	0.0172	(0.005, 0.06)	5.86	(4.06, 7.66)	12.1	0.0077	(0.0019, 0.0305)	7.03	(5.04, 9.02)	15.8
miR-125b-5p	0.011	(0.0033, 0.0374)	6.5	(4.74, 8.26)	11.4	0.0049	(0.0014, 0.0171)	7.67	(5.87, 9.47)	12.1
miR-130a	0.0482	(0.0204, 0.1139)	4.37	(3.13, 5.61)	5.6	0.0215	(0.0067, 0.0691)	5.54	(3.86, 7.23)	10.4
miR-133a	0.0135	(0.002, 0.0899)	6.21	(3.48, 8.97)	45.0	0.0061	(8e-04, 0.0472)	7.36	(4.4, 10.35)	61.5
miR-139-5p	0.0278	(0.0096, 0.0803)	5.17	(3.64, 6.7)	8.4	0.0124	(0.0034, 0.0451)	6.34	(4.47, 8.2)	13.3
miR-143	0.0066	(0.0014, 0.03)	7.25	(5.06, 9.44)	20.9	0.0029	(7e-04, 0.0116)	8.42	(6.43, 10.41)	15.8
miR-145	0.0632	(0.019, 0.2104)	3.98	(2.25, 5.72)	11.1	0.0281	(0.0079, 0.1)	5.15	(3.32, 6.98)	12.6
miR-146a-5p	2.5125	(1.1996, 5.2624)	-1.33	(-2.4, -0.26)	4.4	1.1177	(0.4602, 2.7145)	-0.16	(-1.44, 1.12)	5.9
miR-146b-5p	0.2664	(0.1446, 0.4907)	1.91	(1.03, 2.79)	3.4	0.1185	(0.0527, 0.2664)	3.08	(1.91, 4.25)	5.1
miR-16	5.7065	(3.4326, 9.4867)	-2.51	(-3.25, -1.78)	2.8	2.5385	(0.9094, 7.0863)	-1.34	(-2.83, 0.14)	7.8
miR-17-5p	2.0096	(1.3844, 2.9171)	-1.01	(-1.54, -0.47)	2.1	0.8939	(0.2775, 2.8797)	0.16	(-1.53, 1.85)	10.4
miR-191	5.2304	(2.5084, 10.9061)	-2.39	(-3.45, -1.33)	4.4	2.3267	(0.9523, 5.6846)	-1.22	(-2.51, 0.07)	6.0
miR-193a-5p	0.0272	(0.0063, 0.1182)	5.2	(3.08, 7.32)	18.9	0.0121	(0.0035, 0.0424)	6.37	(4.56, 8.17)	12.2
miR-19b-3p	3.871	(2.8714, 5.2185)	-1.95	(-2.38, -1.52)	1.8	1.722	(0.6496, 4.5646)	-0.78	(-2.19, 0.62)	7.0
miR-20a-5p	0.8846	(0.7015, 1.1155)	0.18	(-0.16, 0.51)	1.6	0.3935	(0.1356, 1.142)	1.35	(-0.19, 2.88)	8.4
miR-20b	0.2363	(0.1897, 0.2943)	2.08	(1.76, 2.4)	1.6	0.1051	(0.0341, 0.3243)	3.25	(1.62, 4.88)	9.5
miR-21	0.2715	(0.1277, 0.5772)	1.88	(0.79, 2.97)	4.5	0.1208	(0.0508, 0.287)	3.05	(1.8, 4.3)	5.6
miR-221-3p	0.3591	(0.1389, 0.9283)	1.48	(0.11, 2.85)	6.7	0.1597	(0.0554, 0.4604)	2.65	(1.12, 4.17)	8.3
miR-23a-3p	0.0143	(0.004, 0.0516)	6.12	(4.28, 7.97)	13.0	0.0064	(0.0022, 0.0187)	7.29	(5.74, 8.85)	8.6
miR-24	4.247	(2.0415, 8.8354)	-2.09	(-3.14, -1.03)	4.3	1.8893	(0.8243, 4.3304)	-0.92	(-2.11, 0.28)	5.3
miR-26b	0.0325	(0.0131, 0.0803)	4.94	(3.64, 6.25)	6.1	0.0144	(0.0038, 0.0547)	6.11	(4.19, 8.04)	14.4
miR-28-5p	0.0133	(0.0049, 0.0358)	6.23	(4.8, 7.66)	7.3	0.0059	(0.0018, 0.0192)	7.4	(5.7, 9.1)	10.5
miR-29a-3p	0.0361	(0.0071, 0.183)	4.79	(2.45, 7.13)	25.7	0.0161	(0.0041, 0.0626)	5.96	(4, 7.92)	15.2
miR-29c-3p	0.0064	(0.0019, 0.0212)	7.29	(5.56, 9.04)	11.1	0.0028	(9e-04, 0.0084)	8.47	(6.89, 10.05)	9.0
miR-301	0.0104	(0.0037, 0.0289)	6.59	(5.11, 8.07)	7.8	0.0046	(0.0011, 0.0194)	7.76	(5.69, 9.83)	17.7
miR-335-5p	0.0045	(9e-04, 0.0232)	7.79	(5.43, 10.16)	26.5	0.002	(3e-04, 0.0121)	8.96	(6.37, 11.56)	36.7
miR-339-3p	0.0048	(0.0013, 0.0178)	7.69	(5.81, 9.57)	13.6	0.0022	(5e-04, 0.009)	8.86	(6.79, 10.92)	17.5
miR-339-5p	0.0252	(0.0079, 0.0803)	5.31	(3.64, 6.98)	10.2	0.0112	(0.003, 0.042)	6.48	(4.57, 8.39)	14.1
miR-375	0.0356	(0.0056, 0.2273)	4.81	(2.14, 7.49)	40.9	0.0158	(0.0027, 0.0921)	5.98	(3.44, 8.52)	33.9
miR-451	1.9405	(0.9182, 4.1009)	-0.96	(-2.04, 0.12)	4.5	0.8632	(0.2735, 2.7245)	0.21	(-1.45, 1.87)	10.0
miR-483-5p	0.5978	(0.1829, 1.9537)	0.74	(-0.97, 2.45)	10.7	0.2659	(0.0861, 0.8213)	1.91	(0.28, 3.54)	9.5
miR-484	4.9561	(2.5401, 9.6701)	-2.31	(-3.27, -1.34)	3.8	2.2047	(0.7225, 6.728)	-1.14	(-2.75, 0.47)	9.3
miR-92	2.393	(1.2048, 4.7529)	-1.26	(-2.25, -0.27)	3.9	1.0645	(0.3843, 2.9487)	-0.09	(-1.56, 1.38)	7.7

**Fig 4:**
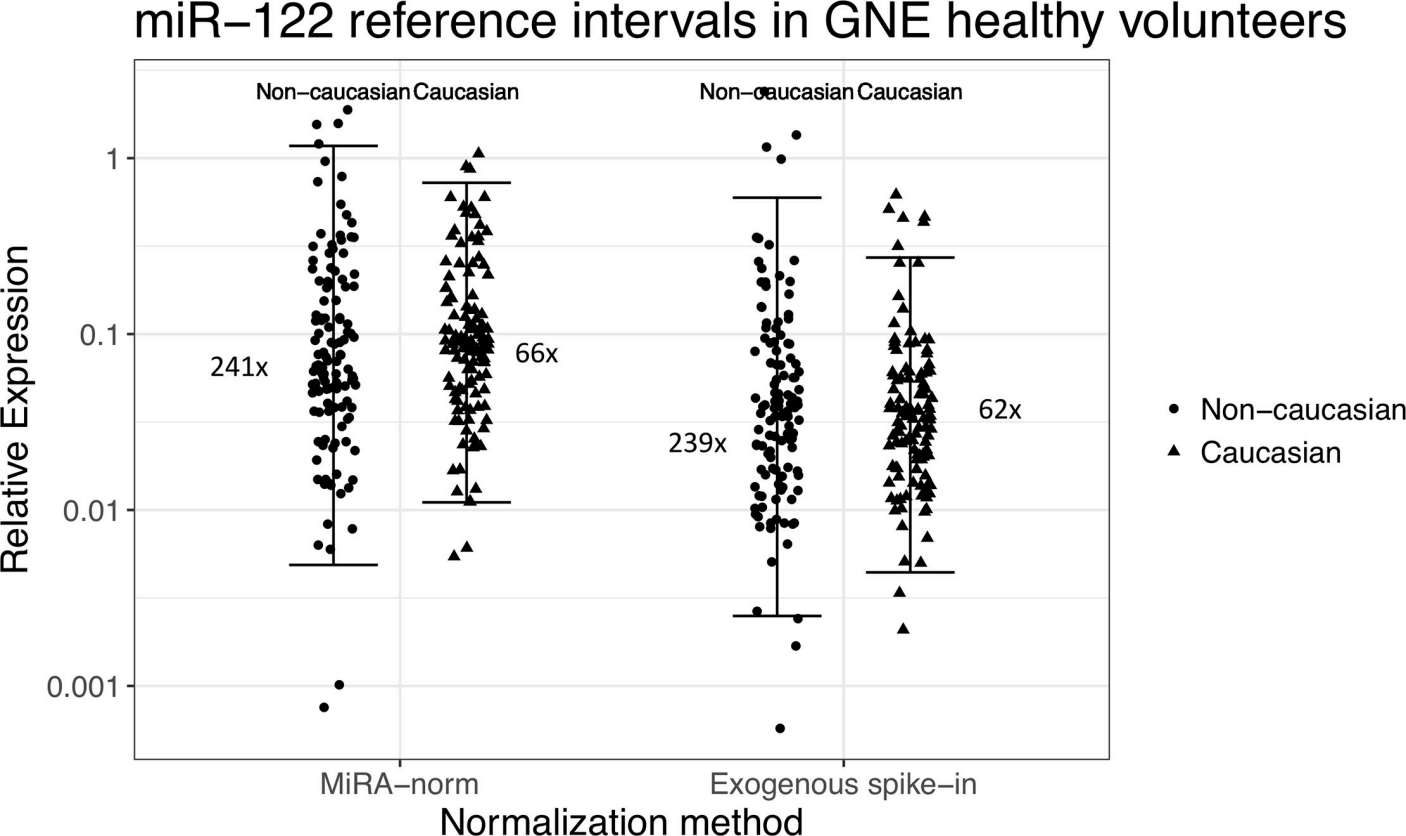
The 95% confidence reference ranges of miR-122 in serum from healthy volunteers separated by individually-identified ethnicity. The relative serum expression of miR-122 normalized to the mean expression of a panel of five endogenous miRNAs (“MiRA-norm”, left) or to the exogenous *C eleg* miR-39 exogenous spike-in (right) separated out by samples from volunteers that identified as Caucasian (▲) and those that did not (e.g. Non-Caucasian) (●). Brackets represent the 95% confidence reference ranges for miR-122 serum expression normalized to miRNA-norm (left) or to *C eleg* miR-39 expression (right), where miR-122 relative expression spanned 239 to 241- and 62 to 66-fold when for Non-Caucasian and Caucasian ethnicity, respectively.

We next explored whether gender and ethnicity contributed to the high variability observed in circulating miR-122 levels in serum from healthy volunteers. The 40-volunteer cohort was comprised of 60% (24/40) male and 40% (16/40) female (Fig 1). There was no difference in variability of circulating miR-122 in serum between male and female volunteers as measured by the 95% reference intervals (S2 Fig). In contrast, the variability of miR-122 appeared to be in part dependent upon the individually-identified ethnicity of the population. The cohort of volunteers evaluated in this study were comprised of 50% (20/40) of individuals that identify with the ethnicity of Caucasian, while the remaining 50% were comprised of various individually-identified ethnicities (Fig 1). When separating out these two classifications of ethnicity (e.g. Caucasian vs. Non-Caucasian), there was an approximately 3 to 4-fold higher variability in miR-122 expression in serum from Non-Caucasian volunteers in relation to serum from Caucasian-only volunteers, with corresponding 95% reference intervals spanning approximately 240-fold and 60-fold regardless of the normalizing approach, respectively (Fig 4). The effect on self-identified ethnicity on the variability of miR-122 in serum from healthy volunteers also appeared to be miR-122-specific in nature as only one other miRNA (miR-483-5p) demonstrated approximately 3-fold difference in the 95% reference intervals of relative expression between Caucasian and Non-Caucasian populations (S4 Table).

### Variance component analysis of miR-122 and other abundant circulating miRNAs

As circulating miR-122 in human serum from healthy volunteers experienced high variability regardless of normalization approach, we then performed variance components analysis to determine the relative contributions of between-donor and within-donor variance to the total variability of the data from the longitudinal study. Estimates of within- and between- donor variance were derived from the random-effects model that was fit for each assay, and the contribution of each component of variation was calculated by dividing the corresponding component variance by the sum of the variance of the two components (i.e. total variance). To highlight the within-donor changes relative to the total variance, we plotted the relative expression of miR-122 in circulation (S3A and S3B Fig) as well as ALT and AST activity levels (S4A and S4B Fig) as a function of time within a subject.

**Fig 5:**
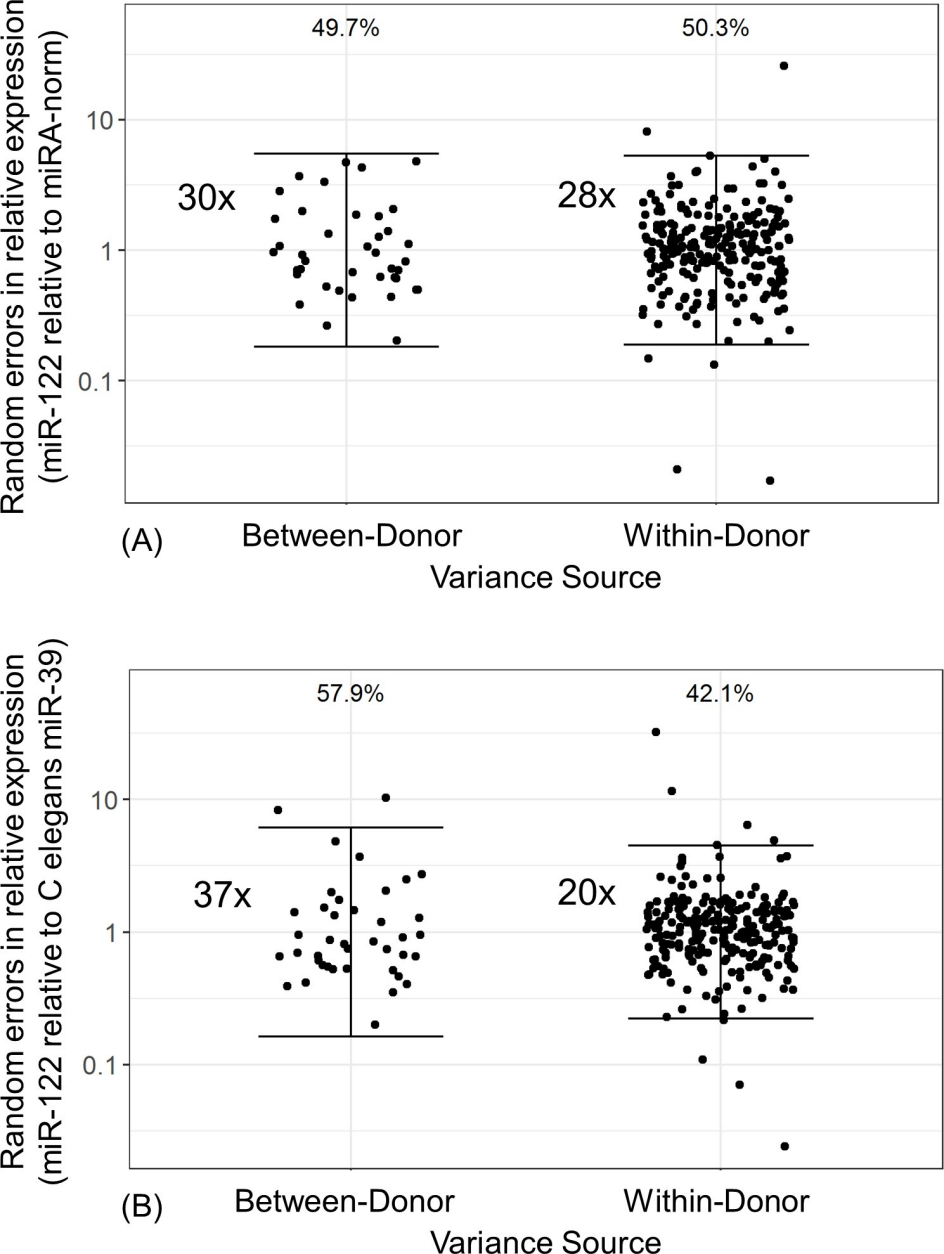
Variance component analysis of miR-122 in serum from healthy volunteers. Variance components of miR-122 relative expression normalized using MiRA-norm (A) or *C*. *eleg* miR-39 (B) in serum from healthy volunteers. Points represent random deviations from between-donor (left) and within-donor (right) means and solid brackets represent reference ranges based on between- (left) and within- (right) donor variation. Proportion of variance relative to the total is displayed above each variance component.

**Fig 6:**
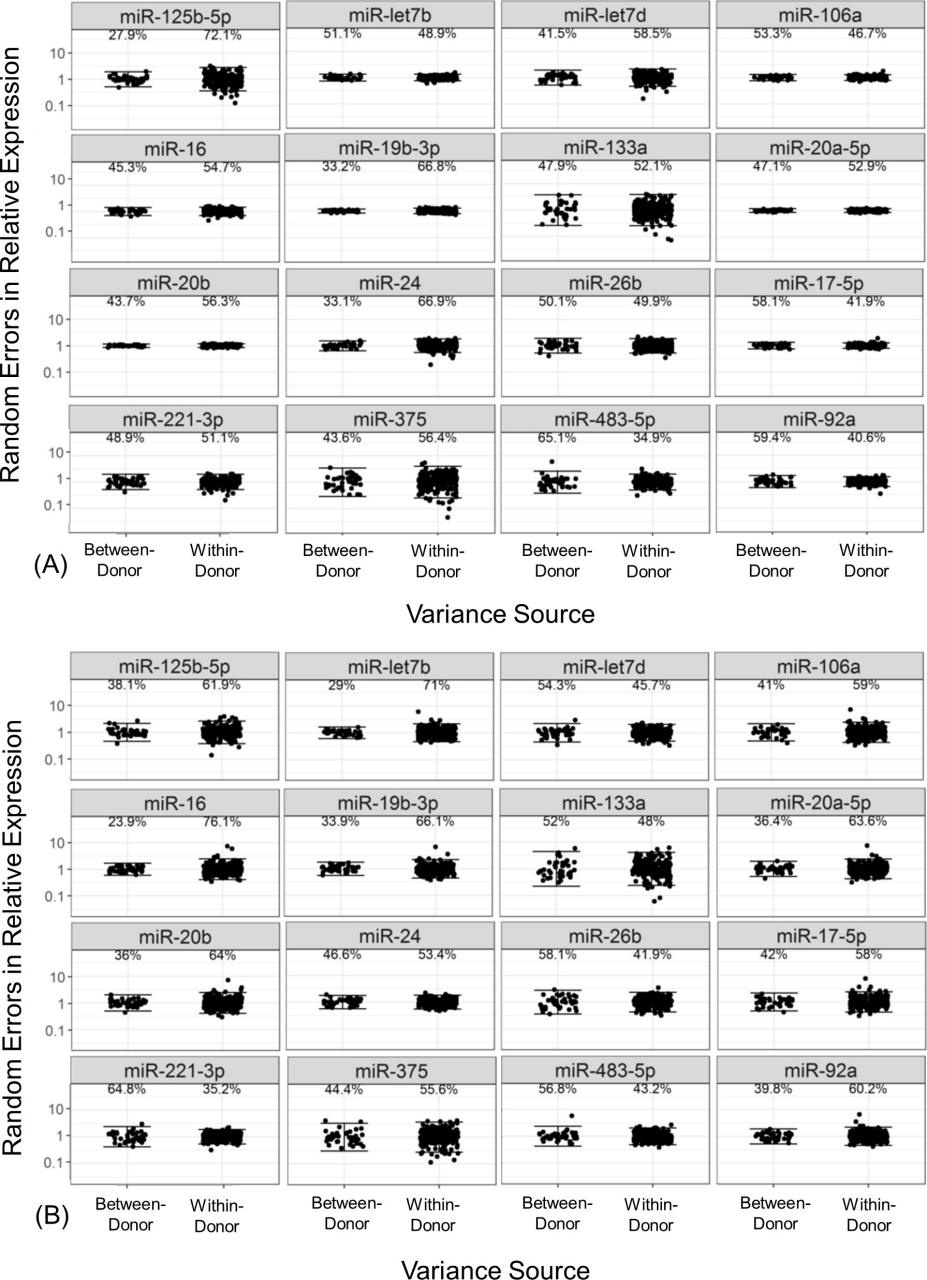
Variance component analysis of the relative expression of a subset of abundant miRNAs in serum from healthy volunteers. Variance component analysis of the relative expression of 16 miRNAs in human serum from healthy volunteers normalized to MiRA-norm (A) or *C*. *eleg* miR-39 (B). Points represent random deviations from between-donor (left) and within-donor (right) means and solid brackets represent 95% reference ranges of each variance component. The overall proportion of variance relative to the total is displayed above each variance component.

**Fig 7:**
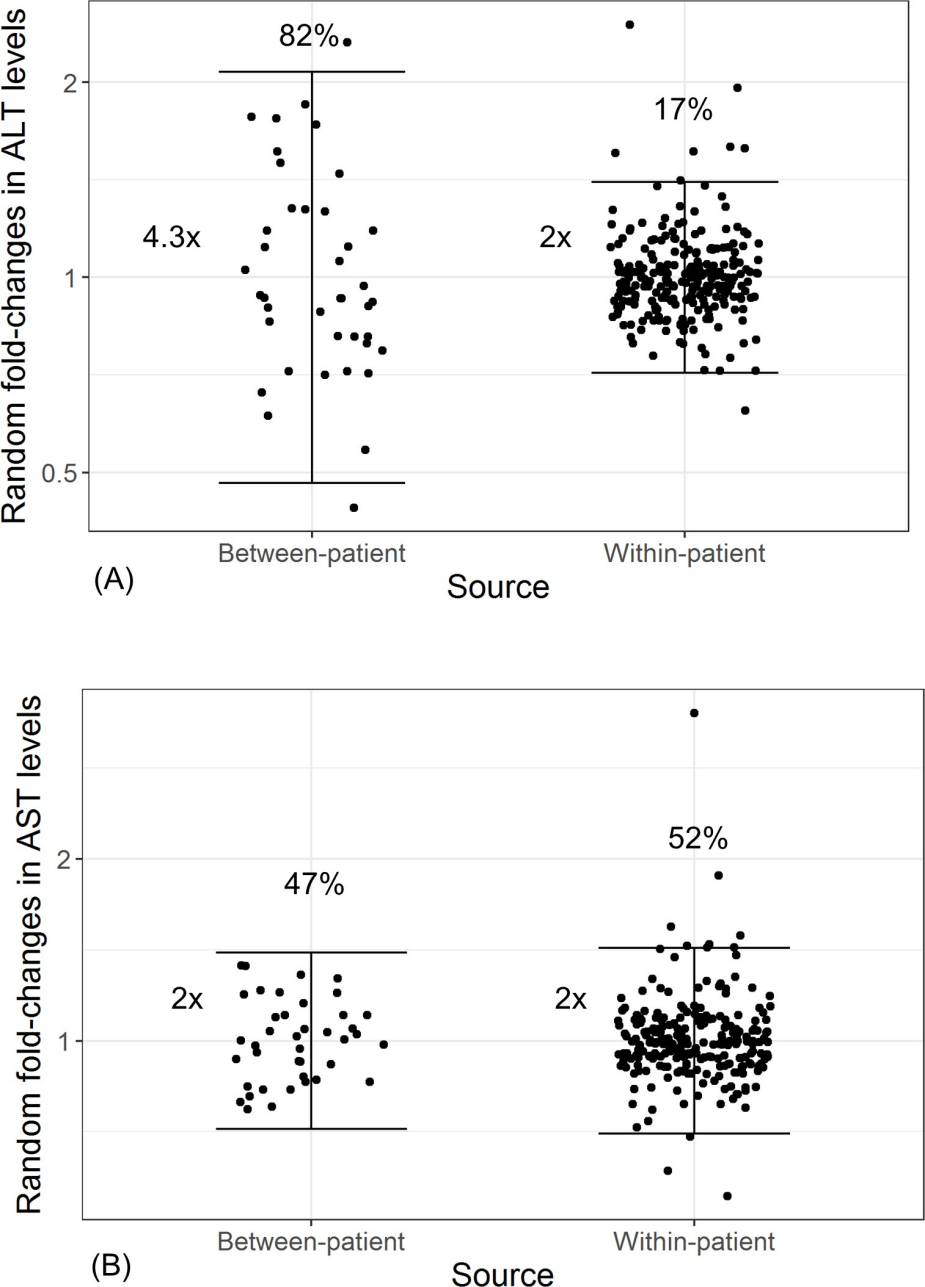
Variance component analysis of liver transaminases in serum from healthy volunteers. Variance component analysis of alanine transaminase (ALT) and aspartate transaminase (AST) are represented in panels A and B, respectively. Points represent random deviations from between-donor (left) and within-donor (right) means and brackets represent 95% reference ranges based of each variance component. Proportion of variance relative to the total is displayed above each variance component.

Variance component analysis on circulating miR-122 in serum from healthy volunteers revealed that the sources of miR-122 total variance were comprised of approximately 50% from within a donor and 50% from between donors, regardless of the normalization strategy employed (Table 3, Fig 5A and 5B). Furthermore, the percent total variance of other well- characterized miRNAs ((such as miR-let7d, miR-16, and miR-92a, among others) [6, 9, 54–57]) in our panel was also relatively evenly split within- and between- donors. However, there were some exceptions (miR-125-5p), which had greater contributions of within-donor variability than between donors (Table 3, Fig 6A and 6B). In comparison, variance analysis of circulating ALT in the identical samples revealed that 80% of variability was derived from between donors, while AST demonstrated variance components similar to miR-122 with roughly 50/50 split between- and within-donors over time (Fig 7A and 7B, Table 4). Lastly, we explored what the correlation of miR-122 relative expression in serum was with ALT and AST in healthy volunteers. Similar to previous reports [24, 25], circulating miR-122 levels in serum from healthy volunteers had positive correlations with serum ALT and AST levels in these healthy volunteers, regardless of the normalization strategy (S5A–S5D Fig).

**Table 3:** Variance components, percent total variance, and fold change of relative expression for a subset of abundant miRNAs in serum from healthy volunteers.

Gene	Source	N	MiRA-norm				*C*. *elegans* miR-39			
			Std. Dev. (Ct)	Variance	% Total Variance	Fold Change Range of Relative Expression	Std. Dev. (Ct)	Variance	% Total Variance	Fold Change Range of Relative Expression
Cel-miR-39	Between-Patient	40	0.43	0.19	34.9	3.6	NA	NA	NA	NA
Cel-miR-39	Within-Patient	6	0.59	0.35	65.1	5.0	NA	NA	NA	NA
Cel-miR-39	Total	240	0.73	0.53	100	7.5	NA	NA	NA	NA
miR-122	Between-Patient	40	1.21	1.47	49.7	30.1	1.29	1.67	57.9	37.5
miR-122	Within-Patient	6	1.22	1.49	50.3	28.1	1.1	1.21	42.1	20.3
miR-122	Total	240	1.72	2.96	100	116.9	1.7	2.88	100	110.7
miR-let7b	Between-Patient	40	0.21	0.05	51.1	1.8	0.36	0.13	29	2.7
miR-let7b	Within-Patient	6	0.21	0.04	48.9	1.8	0.56	0.31	71	4.6
miR-let7b	Total	240	0.3	0.09	100	2.3	0.66	0.44	100	6.2
miR-17-5p	Between-Patient	40	0.2	0.04	58.1	1.8	0.55	0.3	42	4.7
miR-17-5p	Within-Patient	6	0.17	0.03	41.9	1.6	0.65	0.42	58	5.8
miR-17-5p	Total	240	0.27	0.07	100	2.1	0.85	0.72	100	10.4
miR-19b-3p	Between-Patient	40	0.13	0.02	33.2	1.4	0.41	0.17	33.9	3.2
miR-19b-3p	Within-Patient	6	0.18	0.03	66.8	1.6	0.58	0.33	66.1	4.8
miR-19b-3p	Total	240	0.22	0.05	100	1.8	0.71	0.5	100	7.0
miR-20a-5p	Between-Patient	40	0.12	0.01	47.1	1.4	0.47	0.22	36.4	3.7
miR-20a-5p	Within-Patient	6	0.12	0.01	52.9	1.4	0.62	0.38	63.6	5.4
miR-20a-5p	Total	240	0.17	0.03	100	1.6	0.77	0.6	100	8.4
miR-20b	Between-Patient	40	0.11	0.01	43.7	1.3	0.49	0.24	36	4.0
miR-20b	Within-Patient	6	0.12	0.01	56.3	1.4	0.65	0.43	64	6.0
miR-20b	Total	240	0.16	0.03	100	1.6	0.82	0.67	100	9.5
miR-133a	Between-Patient	40	0.95	0.91	47.9	14.4	1.07	1.15	52	20.3
miR-133a	Within-Patient	5.7	0.99	0.99	52.1	15.1	1.03	1.06	48	16.8
miR-133a	Total	228	1.38	1.89	100	45.0	1.49	2.21	100	61.5
miR-let7d	Between-Patient	40	0.46	0.21	41.5	3.7	0.57	0.32	54.3	4.9
miR-let7d	Within-Patient	6	0.55	0.3	58.5	4.5	0.52	0.27	45.7	4.2
miR-let7d	Total	240	0.72	0.52	100	7.3	0.77	0.6	100	8.5
miR-106a	Between-Patient	40	0.2	0.04	53.3	1.7	0.53	0.28	41	4.4
miR-106a	Within-Patient	6	0.19	0.03	46.7	1.7	0.64	0.41	59	5.7
miR-106a	Total	240	0.27	0.07	100	2.1	0.83	0.69	100	10.0
miR-125b-5p	Between-Patient	40	0.47	0.22	27.9	3.7	0.56	0.31	38.1	4.8
miR-125b-5p	Within-Patient	6	0.75	0.57	72.1	7.8	0.71	0.51	61.9	7.0
miR-125b-5p	Total	240	0.89	0.78	100	11.4	0.9	0.82	100	12.1
miR-130a	Between-Patient	40	0.33	0.11	28.7	2.6	0.62	0.39	54.3	5.7
miR-130a	Within-Patient	6	0.53	0.28	71.3	4.2	0.57	0.33	45.7	4.8
miR-130a	Total	240	0.62	0.39	100	5.6	0.84	0.71	100	10.4
miR-16	Between-Patient	40	0.25	0.06	45.3	2.0	0.37	0.13	23.9	2.8
miR-16	Within-Patient	6	0.27	0.07	54.7	2.1	0.65	0.42	76.1	5.9
miR-16	Total	240	0.37	0.14	100	2.8	0.75	0.56	100	7.8
miR-24	Between-Patient	40	0.31	0.09	33.1	2.4	0.41	0.17	46.6	3.2
miR-24	Within-Patient	6	0.44	0.19	66.9	3.3	0.44	0.19	53.4	3.3
miR-24	Total	240	0.53	0.28	100	4.3	0.6	0.36	100	5.3
miR-26b	Between-Patient	40	0.46	0.21	50.1	3.7	0.73	0.54	58.1	7.8
miR-26b	Within-Patient	6	0.46	0.21	49.9	3.5	0.62	0.39	41.9	5.5
miR-26b	Total	238	0.65	0.43	100	6.1	0.96	0.93	100	14.4
miR-221-3p	Between-Patient	40	0.48	0.23	48.9	3.9	0.61	0.38	64.8	5.6
miR-221-3p	Within-Patient	6	0.49	0.24	51.1	3.8	0.45	0.2	35.2	3.4
miR-221-3p	Total	240	0.69	0.47	100	6.7	0.76	0.58	100	8.3
miR-375	Between-Patient	40	0.89	0.79	43.6	12.0	0.85	0.72	44.4	10.8
miR-375	Within-Patient	6	1.01	1.02	56.4	16.0	0.95	0.91	55.6	13.5
miR-375	Total	240	1.34	1.81	100	41.0	1.28	1.63	100.0	34.0
miR-483-5p	Between-Patient	40	0.69	0.47	65.1	6.9	0.61	0.38	56.8	5.6
miR-483-5p	Within-Patient	6	0.5	0.25	34.9	4.0	0.53	0.29	43.2	4.3
miR-483-5p	Total	240	0.85	0.73	100	10.7	0.81	0.66	100	9.5
miR-92a	Between-Patient	40	0.38	0.15	59.4	2.9	0.47	0.22	39.8	3.7
miR-92a	Within-Patient	6	0.32	0.1	40.6	2.4	0.57	0.33	60.2	4.8
miR-92a	Total	240	0.49	0.24	100	3.9	0.74	0.55	100	7.7

**Table 4:** Variance component analysis of alanine transaminase (ALT) and aspartate transaminase (AST) in serum from healthy volunteers.

Transaminase	Source	Std. Dev (log2)	Variance	% Total Variance	Fold change range (+/- 2 SD)
ALT	Between-patient	0.53	0.28	82.3	4.3
	Within-patient	0.24	0.06	17.7	2
	Total	0.58	0.34	100	5
AST	Between-patient	0.24	0.06	47.4	2
	Within-patient	0.26	0.07	52.6	2
	Total	0.35	0.12	100	2.7

## Discussion

As of 2019, over 3800 miRNA species have been identified in humans (miRBase v.22.1 October 2018). Circulating miRNAs are attractive candidates for biomarker development due to their relative specificity, as well as their accessibility and stability in biological fluids [58]. Specifically, miR-122 has been proposed to be a promising biomarker of liver function, health, and disease due to its organ-specific expression. Additionally, it has been reported that increases in circulating levels may be associated with a wide variety of liver dysfunctions, ranging from acute to chronic conditions [24, 31, 37]. However, these associations were primarily based upon differences in population means between a given disease state and healthy or other controls [25–27]. To date, little is known about the baseline variability of circulating levels of miR-122 in healthy humans, and in particular, the components of this variance that affect the practical use of the biomarker in the clinic. In part, this is due to the lack of dedicated studies designed to assess variability of this exploratory biomarker in humans, as well as a lack of a standardized method to measure and report circulating levels of miRNAs in serum or plasma. Thus, the goal of this study was to measure the total variance and the contributions of inter- and intra-subject variability of circulating levels of miR-122 in a cohort of healthy volunteers across a longitudinal study.

The major finding in this report was the remarkably high variance of circulating levels of miR-122 in serum from healthy volunteers. In particular, the relative miR-122 expression in serum from healthy volunteers had 95% reference intervals that spanned an interval of >100-fold, regardless of normalization method (Fig 2A). In the same cohort of healthy volunteers, traditional transaminase activities had 95% reference intervals that spanned between 2 and 5- fold (Fig 2B). The high variance of miR-122 is in line with recently published work that used an absolute quantitative approach for detecting miR-122 in healthy volunteers from two separate cohorts [25]. In this cited publication, circulating levels of miR-122 demonstrated 95% reference intervals that spanned 40- and 75-fold around the mean levels. It is not clear at this point if the analysis methods or subject samples are responsible for the differences in 95% reference intervals between our findings, and that of this recent article. However, both studies support high variance of this biomarker in healthy human subjects, especially in light of traditionally employed liver transaminases. However, the high degree of overall total variability appeared to be somewhat miR-122-specific: it had nearly 2-fold larger reference intervals than the next most variable miRNA in circulation for the panel of miRNAs examined here, regardless of normalization approaches (Table 2, Fig 4A and 4B). This novel finding implied that there are factors specific to miR-122 biology, stability in circulation, or other processes resulting in the high variability in healthy volunteers that was not observed for 30 other high abundant miRNAs in this population.

Here, we also report that the high total variance of miR-122 relative expression in serum of healthy volunteer subjects was comprised of approximately equivalent components of inter- and intra- subject variability (Fig 6A and 6B), a finding that is also in concordance with Church et al. (2019). In other words, miR-122 can fluctuate up to 28-fold within the 95% reference interval for this component within a donor. Similar to miR-122, the approximately equivalent variance components were also observed with other high-abundant miRNAs (Table 3, Fig 6A and 6B) and thus does not appear to be miR-122 specific. While the levels of miR-122 in serum was correlated with ALT and AST in healthy volunteers (S5 Fig) with moderate Pearson r values similar to those reported with previous reports [24, 25], there were significant difference in the variance components between miR-122 and transaminases. In contrast, approximately 80% of variance of ALT was derived from between donors (Table 4). Taken together, we believe further investigation is needed to fully understand biological sources of variability of miR-122 in circulation.

A major challenge we faced with this study was the uncertainty regarding the appropriate experimental and analytical approaches to use to measure circulating miR-122 in human serum. Currently there is not a standard and generally well-accepted experimental, analytical, and normalization guidance for detecting and quantifying miRNAs in biofluids. As such, we decided to employ a qRT-PCR-based relative quantification assay with two different normalization approaches to measure circulating miR-122 and the other abundant miRNAs in human serum. In particular, we normalized miR-122 levels to an exogenous “spike in” control (*C eleg* miR-39) for controlling the technical variability that may be introduced at different sample handling, processing, and analysis steps. This approach, although commonly employed [27, 42], only controls for RNA purification, reverse transcription efficiencies, and other sample processing steps and not for the initial quality of samples that may have various levels of global miRNA degradation caused by disease biology, cell lysis, or other unknown factors. Another common normalization method is to report the expression of the miRNA of interest relative to preselected “endogenous-like” miRNAs that appear both abundant and stably expressed in circulation. This approach can introduce bias into the analysis, as it is not always clear what factors affect the circulating levels of these preselected endogenous miRNAs. This is particularly important when evaluating circulating miRNA samples from different populations, including patients with varying disease states. For example, miR-16 has been published as an appropriate endogenous miRNA for normalization [42, 59]; yet has been shown to be downregulated in malignant prostate cancer tissues [60] and upregulated in women with breast cancer [61].

To overcome the potential challenges of using individual preselected endogenous miRNAs for normalization, we employed a novel, unbiased approach for selecting appropriate endogenous circulating miRNAs for normalization (e.g. MiRA-norm). This method used an algorithm that identifies miRNAs that move in coordination across the entire sample set [48]. A limitation of the MiRA-norm approach is that its accuracy depends on the number of miRNAs assessed per sample and the number of samples analyzed in the data set. As such, this approach does not appear appropriate for small sample sets with a handful of targeted miRNAs being profiled. In order to ensure robust and accurate selection of endogenous miRNA using MiRA-norm, we analyzed a diverse panel of 80 miRNAs for >200 serum samples. This not only allowed for accurate endogenous miRNAs to be selected, but also for comparing the variance of miR-122 in circulation to other highly abundant miRNA in circulation. In this report, the MiRA-norm method selected 5 miRNAs to employ as endogenous-like miRNAs for normalization. Not surprisingly, this panel was selected consistently across 4 other human serum sample sets using the identical miRNA panel and experimental conditions (data not shown), supporting that this method can consistently identify stably expressed miRNAs in human serum. While further assessment of the biological variance of these 5 miRNAs broadly across different disease states and populations is needed, it is possible that repeated use of the MiRA-norm method could identify stable miRNAs to use for preselected normalization approaches. However, there is a more pressing need to establish global standard procedures for assessing miRNAs in biofluids, enabling consistency across the field, and the ability to directly compare findings across studies. This will facilitate more efficient evaluation and qualification of miR-122 and/or other promising miRNA biomarkers in both nonclinical and clinical settings.

In conclusion, we believe the variable nature, coupled with different biological etiologies which are postulated to affect miR-122 expression, may make this miRNA challenging for use as a prospective clinical monitoring tool. This inference was based solely on the overall variance of miR-122 in serum from healthy volunteers and not on data from clinical samples of suspected liver injury and/or disease. As outlined in recent reports [25, 26], there likely are specific contexts of use that in miR-122 could fulfill and considerations for these areas are out of scope for this work. The probability of clinical success with this biomarker as a prospective monitoring tool is dependent upon a rigorous understanding on its movement to identify upper bounds of expression in normal healthy individuals. This report provides foundational data in this regard. However, further studies are needed using standardized experimental and analytical approaches to better identify clinical departure points (e.g. a meaningful fold change from normal). It is important to note that an in-depth analysis of other factors which may affect miR-122 expression such as biological or circadian rhythms and other chronic disease states such as diabetes were not addressed in this study [13, 18, 19, 62]. In addition, we cannot rule out the possibility that undiagnosed and underlying liver diseases, such as NAFLD, contributed to the high variability of miR-122 in circulation in this study. This study was also not designed to address age-related effects on miR-122 levels in circulation. However, all volunteers were between the ages of 20 and 65 years old at time of blood donations, and as such represent the appropriate adult population that would be encountered clinically for most indications. In addition, it remains unknown the role that the time of blood draws have on the variability of miR-122 levels in serum from healthy volunteers observed in this study. However, all blood donations were consistently performed in the morning between the hours of 7 and 10 am when possible (S1 Table). No restrictions on food consumption were implemented and accordingly some of the variability in circulating miR-122 levels in serum may be due in part do the time of the day samples were collected and food-related effects.

While we did not observe notable contributions from gender which could have explained the high variance of miR-122 (S2 Fig), we did find that ethnicity may have played a role in this higher than expected variability that was observed. In particular, it appears that individuals that identify as Caucasian have lower miR-122 serum variability than Non-Caucasian individuals, where miR-122 levels in serum from donors that identified as non-Caucasian experienced 3 to 4-fold larger reference internals than observed in serum from donors that identified as Caucasian (Fig 4, S4 Table). These findings are also in concordance with that of Church et. al. 2019, which observed higher variability of circulating miR-122 in samples from individuals that identified as black relative to those that identified as white (e.g. Caucasian) [25]. Interestingly, the effect we observed on ethnicity appears to be relatively miR-122 specific, as only miR-122 and miR-486-5p of 19 abundant miRNAs assessed demonstrated >3-fold difference in 95% reference interval between Non-Caucasian and Caucasian donors (S4 Table). However, the small sample size (e.g. approximately N = 20 or less per group) likely limits the ability to make statistically significant conclusions in this regard and further studies are warranted to understand the role that ethnicity plays in miR-122 normal expression in circulation.

Expression differences between protein-bound and vesicle/exosome-encapsulated miRNAs were also not explored, nor did we investigate matrix dependent effects (e.g. plasma vs. serum) in this study. These complexities are worthy of further inquiry as the miRNA field and the understanding of these molecules as potential biomarkers continues to expand. Moreover, we cannot exclude the role that miR-122 stability at -80°C plays in the observed variability from this study. In this report, care was taken to ensure all samples were processed for downstream RNA analysis within 6 months of collection and only experienced 1 freeze-thaw cycle. However, it is likely that freezer stability played a minimal role in the higher than expected variability observed and conclusions drawn here based on the overall reported stability of miRNAs in frozen plasma and serum [10] and the consistent findings of high variability regardless of normalizing to endogenous or exogenous miRNAs. Lastly, other exploratory biomarkers of liver injury have emerged recently that appear equivalently sensitive and specific to detect liver injury as miR-122, while not suffering from the high variability in healthy volunteers and technical/analytical challenges of miR-122. In particular, circulating glutamate dehydrogenase (GLDH) has been reported to be a liver specific biomarker of hepatic injury in humans with acceptable variance in healthy volunteers [25, 63]. These emerging clinical monitoring tools are beginning to fulfill some of the contexts of use initially proposed for circulating miR-122. As these gain regulatory acceptance and qualification, the focus on miR-122 to cover similar contexts of use will likely diminish.

## Supporting information

S1 Fig Movement of the miRNA relative expression across a representative sample set of serum from healthy volunteers with emphasis on *C eleg* miR-39, MiRA-norm selected miRNAs, and the mean expression of the 5 selected controls employed by MiRA-norm. miRNAs noted in sidebar are normalizing miRNAs found in common across experiments in human and preclinical models. Using this method, we have identified a fixed panel of endogenous miRNAs for normalization in this study.
(PDF)

S2 Fig Longitudinal movement of miR-122 in serum samples from healthy volunteers. The expression of miR-122 plotted per individual over 6 samples collected over 5–10 weeks relative to MiRA-norm and *C eleg* miR-39 are represented in panels A and B, respectively. For each plot, individual samples are connected by solid lines across the 40 healthy volunteers enrolled in the study. Dashed lines represent 95% reference intervals established from complete data set (N = 240 samples).
(PDF)

S3 Fig The 95% confidence reference ranges of circulating miR-122 levels in serum from healthy volunteers separated by gender. The relative expression of miR-122 in serum from female (●) and male (▲) donors normalized to the mean expression of a panel of five endogenous miRNAs (“MiRA-norm”, left) or to the exogenous *C eleg* miR-39 exogenous spike-in (right). Brackets represent the 95% confidence reference ranges for miR-122 serum expression, where miR-122 relative expression spanned 153- and 104-fold when normalizing to miRNA-norm for serum samples from female and male donors, respectively. Similarly, brackets represent the 95% confidence reference ranges for miR-122 serum expression, where miR-122 relative expression spanned 89- and 133-fold when normalizing to *C eleg* miR-39 exogenous spike-in for serum samples from female and male donors, respectively.
(PDF)

S4 Fig Longitudinal movement of ALT and AST in serum samples from healthy volunteers. Serum levels of ALT and AST plotted per individual over 6 samples collected over 5–10 weeks are represented in panels A and B, respectively. For each plot, individual samples are connected by solid lines across the 40 healthy volunteers enrolled in the study. Dashed lines represent the upper limit of normal for ALT and AST.
(PDF)

S5 Fig Correlations between miR-122 and transaminases. miR-122 normalized to miRA-norm plotted with ALT (A) and AST (B). miR-122 normalized to *C eleg* miR-39 plotted with ALT (C) And AST (D). N = 240.
(PDF)

S1 Table Time of each blood collection for each healthy volunteer visit. (PDF)

S2 Table Gender, age, and transaminase (ALT and AST) levels for each serum sample donated per healthy volunteer (N = 240). (PDF)

S3 Table Panel of miRNAs included in our exploratory panel, with percentage of useable/detectable data across all samples tested. (PDF)

S4 Table Reference interval (95% CI) reported as fold-change of expression for miR-122 expression in serum from volunteers that identified as Caucasian, Non-Caucasian, and total volunteer cohort when expression was normalized using miRA-norm or to *C eleg* miR-39. (PDF)
