# Supplementary material for: Variance component analysis of circulating miR-122 in serum from healthy human volunteers
Source: PLoS One. 2019 Jul 26;14(7):e0220406. doi: 10.1371/journal.pone.0220406 (PMC6660082; doi:10.1371/journal.pone.0220406)

Fig S1. Movement of the miRNA relative expression across a representative sample set of serum from healthy volunteers with emphasis on Cel-miR-39, miRA-norm selected miRNAs, and the mean expression of the 5 selected controls employed by miRA-norm. miRNAs noted in sidebar are normalizing miRNAs found in common across experiments in human and preclinical models. Using this method, we have identified a fixed panel of endogenous miRNAs for normalization in this study.

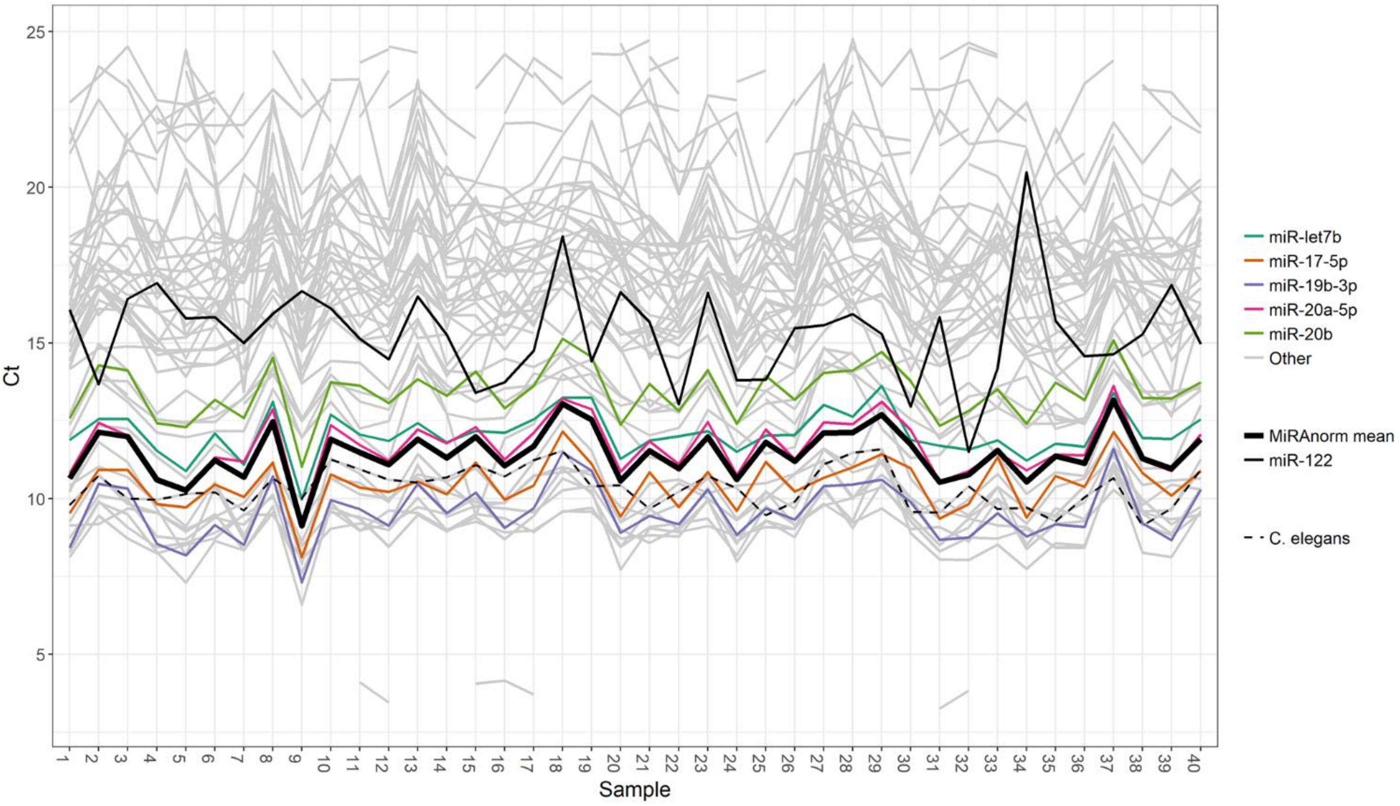

Supplement: S1 Fig — miRNAs noted in sidebar are normalizing miRNAs found in common across experiments in human and preclinical models. Using this method, we have identified a fixed panel of endogenous miRNAs for normalization in this study. (PDF) [file pone.0220406.s001.pdf]
