# Supplementary material for: Variance component analysis of circulating miR-122 in serum from healthy human volunteers
Source: PLoS One. 2019 Jul 26;14(7):e0220406. doi: 10.1371/journal.pone.0220406 (PMC6660082; doi:10.1371/journal.pone.0220406)
Supplement: S3 Fig — The relative expression of miR-122 in serum from female (●) and male (▲) donors normalized to the mean expression of a panel of five endogenous miRNAs (“MiRA-norm”, left) or to the exogenous C eleg miR-39 exogenous spike-in (right). Brackets represent the 95% confidence reference ranges for miR-122 serum expression, where miR-122 relative expression spanned 153- and 104-fold when normalizing to miRNA-norm for serum samples from female and male donors, respectively. Similarly, brackets represent the 95% confidence reference ranges for miR-122 serum expression, where miR-122 relative expression spanned 89- and 133-fold when normalizing to C eleg miR-39 exogenous spike-in for serum samples from female and male donors, respectively. (PDF) [file pone.0220406.s003.pdf]

Fig S3. Longitudinal movement of miR-122 in serum samples from healthy volunteers. The expression of miR-122 plotted per individual over 6 samples collected over 5-10 weeks relative to MiRA-norm (A) and *C eleg* miR-39 (B). Dashed lines represent 95% reference intervals established from complete data set (N=240 samples) for miRNA.

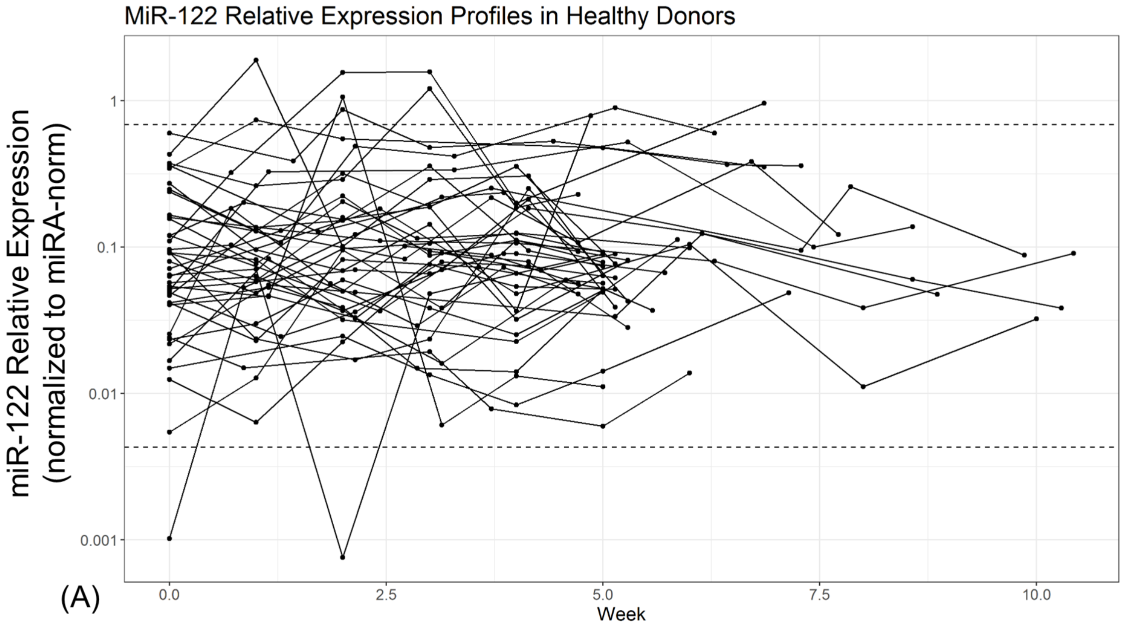

(A)

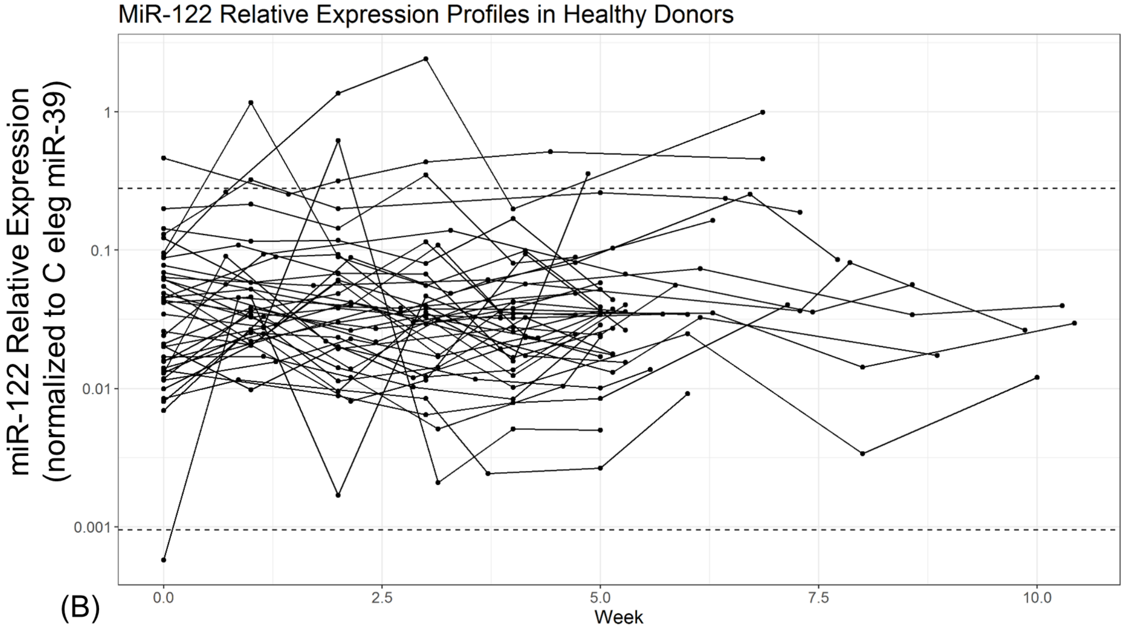

(B)
