# Supplementary material for: Variance component analysis of circulating miR-122 in serum from healthy human volunteers
Source: PLoS One. 2019 Jul 26;14(7):e0220406. doi: 10.1371/journal.pone.0220406 (PMC6660082; doi:10.1371/journal.pone.0220406)
Supplement: S4 Fig — Serum levels of ALT and AST plotted per individual over 6 samples collected over 5–10 weeks are represented in panels A and B, respectively. For each plot, individual samples are connected by solid lines across the 40 healthy volunteers enrolled in the study. Dashed lines represent the upper limit of normal for ALT and AST. (PDF) [file pone.0220406.s004.pdf]

Fig S4. Longitudinal movement of ALT and AST in serum samples from healthy volunteers. The serum levels of ALT (A) and AST (B) plotted per individual over 6 samples collected over 5-10 weeks. For each plot, individual samples are connected by solid lines across the 40 healthy volunteers enrolled in the study. Dashed lines represent the upper limit of normal for ALT and AST.

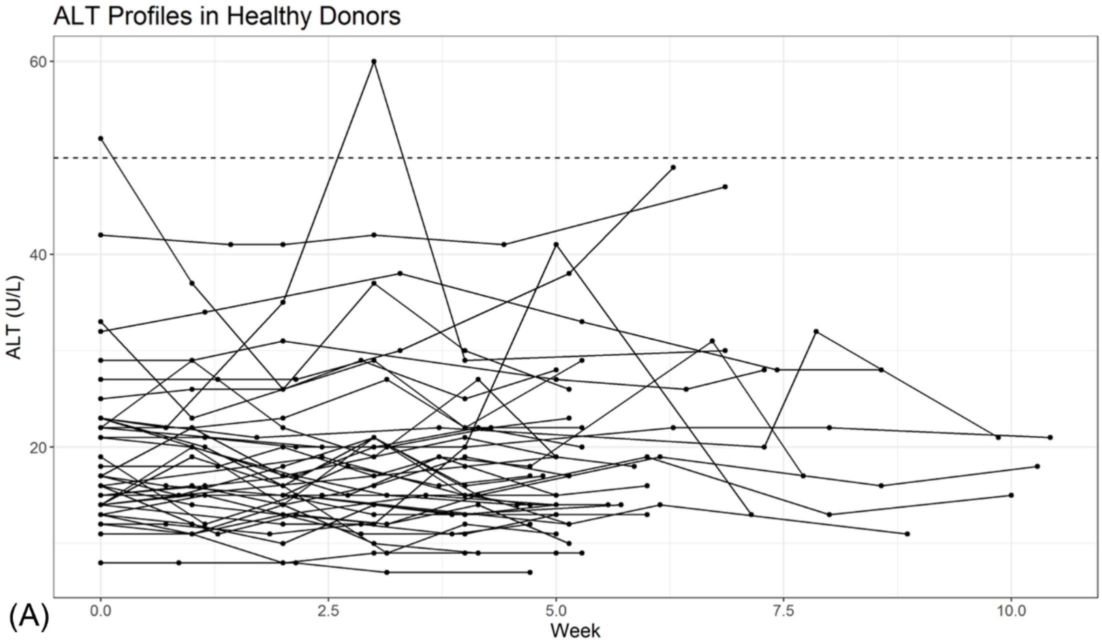

(A)

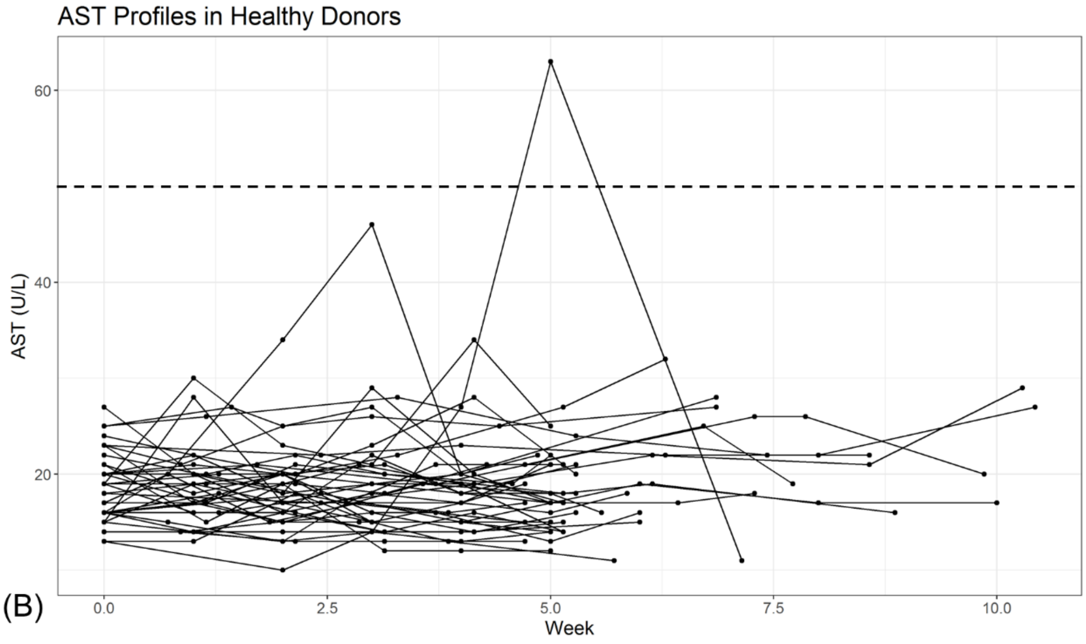

(B)
