# Supplementary material for: Variance component analysis of circulating miR-122 in serum from healthy human volunteers
Source: PLoS One. 2019 Jul 26;14(7):e0220406. doi: 10.1371/journal.pone.0220406 (PMC6660082; doi:10.1371/journal.pone.0220406)

Fig S5. Correlation between miR-122-relative expression and transaminases levels in serum from healthy volunteers. miR-122 normalized to miRA-norm plotted with ALT (A) and AST (B). miR-122 normalized to *C eleg* miR-39 plotted with ALT (C) And AST (D). N=240 with Pearson r coefficient represented on each panel.

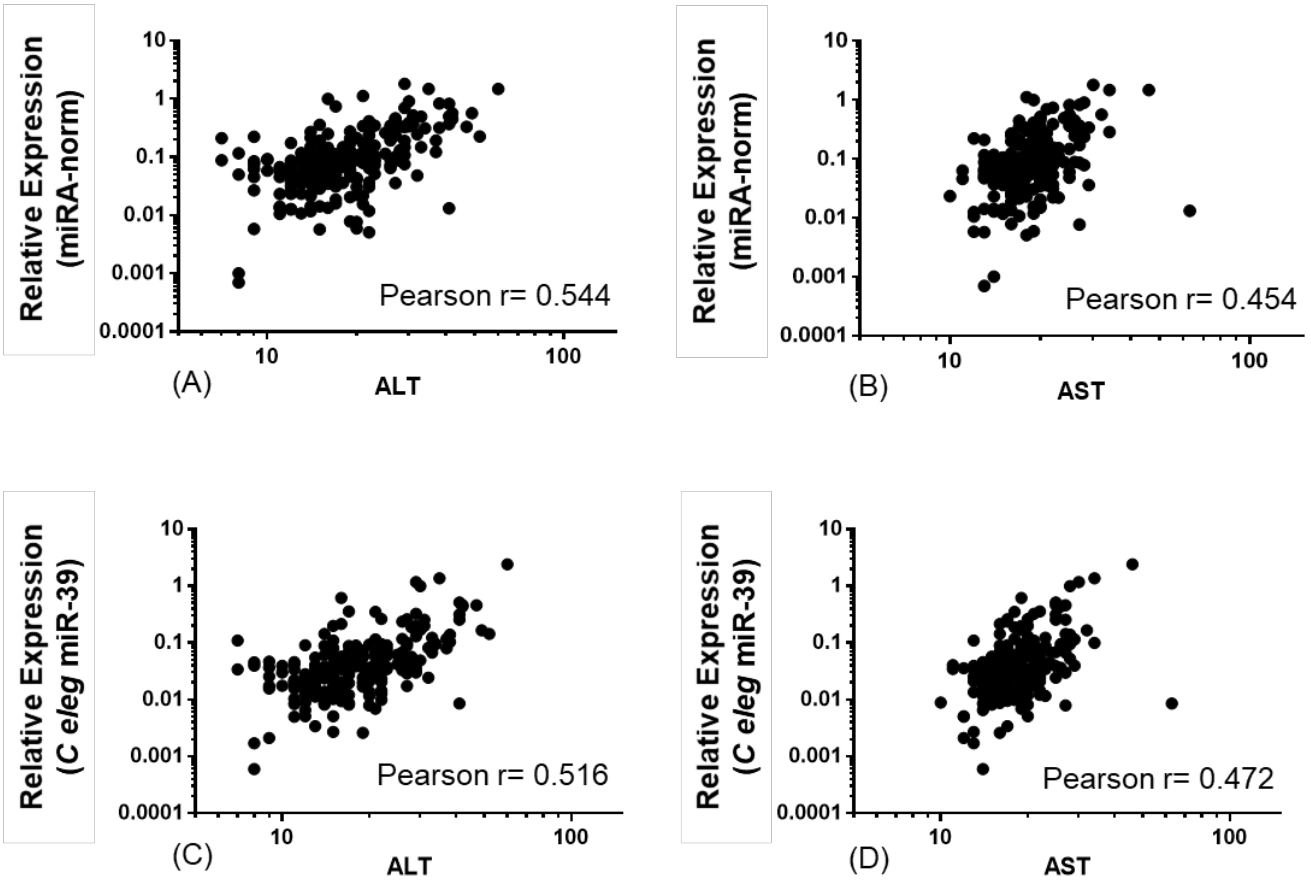

Supplement: S5 Fig — miR-122 normalized to miRA-norm plotted with ALT (A) and AST (B). miR-122 normalized to C eleg miR-39 plotted with ALT (C) And AST (D). N = 240. (PDF) [file pone.0220406.s005.pdf]
