# Supplementary material for: Variance component analysis of circulating miR-122 in serum from healthy human volunteers
Source: PLoS One. 2019 Jul 26;14(7):e0220406. doi: 10.1371/journal.pone.0220406 (PMC6660082; doi:10.1371/journal.pone.0220406)
Supplement: S1 Table — (PDF) [file pone.0220406.s006.pdf]

Table S1. Time of each blood collection in the morning for each healthy volunteer visit.

Volunteer ID	Donation 1	Donation 2	Donation 3	Donation 4	Donation 5	Donation 6
	Time [AM]					
V1	8:25	8:30	8:34	8:36	7:52	9:18
V2	7:30	9:46	9:30	9:00	9:35	9:45
V3	9:42	8:00	9:22	9:06	7:57	9:20
V4	8:14	9:28	8:39	8:39	9:09	8:20
V5	9:20	8:34	8:43	9:38	9:17	9:15
V6	8:35	8:30	8:34	8:22	8:32	8:02
V7	8:20	8:00	9:15	8:30	7:55	8:30
V9	8:45	8:30	8:28	8:30	8:25	8:30
V10	9:43	9:24	9:30	9:35	9:48	9:31
V11	8:00	7:56	7:45	7:45	7:57	9:50
V12	9:21	9:30	9:33	9:25	8:40	9:00
V13	8:58	9:08	9:00	9:00	10:00	9:00
V14	10:00	10:45	9:58	10:02	9:54	9:54
V15	9:00	9:36	11:11	11:01	8:06	9:30
V16	8:34	9:00	8:32	8:29	8:32	8:30
V17	10:01	8:48	9:31	9:00	9:00	9:00
V18	9:42	9:48	9:40	9:44	9:47	9:30
V19	8:40	8:42	8:50	7:40	8:40	8:50
V20	9:11	8:30	8:15	8:00	8:08	8:08
V21	9:30	9:47	9:30	9:35	9:31	8:30
V22	7:35	7:30	7:30	7:30	7:26	7:50
V23	8:20	8:20	8:18	8:20	9:20	8:20
V24	9:40	9:30	9:36	10:10	9:45	9:40
V25	8:51	8:30	8:30	8:15	8:14	8:30
V26	9:29	9:35	9:00	10:00	9:30	9:10
V27	9:15	8:30	8:30	9:07	9:37	9:15
V28	8:00	9:55	7:30	8:16	8:10	9:30
V29	8:45	8:55	8:45	8:43	8:40	8:41
V30	7:40	7:51	7:40	7:40	7:40	7:40
V31	7:45	8:50	7:53	8:05	7:55	8:05
V32	9:00	10:15	10:00	10:00	10:15	10:08
V33	9:06	9:40	9:11	9:12	7:56	9:08
V34	7:25	7:45	8:00	8:00	8:00	7:38
V35	8:15	8:15	8:09	8:15	8:16	8:00
V36	8:45	7:55	7:38	8:45	8:45	8:00
V37	7:53	7:53	8:17	7:55	8:10	8:10
V38	10:00	10:00	10:00	8:00	9:16	9:15
V39	8:33	9:30	9:30	9:30	10:00	9:30
V40	11:28	8:26	9:23	8:11	9:06	8:10
V41	8:30	7:40	7:37	8:05	8:15	8:05
