# Supplementary material for: Variance component analysis of circulating miR-122 in serum from healthy human volunteers
Source: PLoS One. 2019 Jul 26;14(7):e0220406. doi: 10.1371/journal.pone.0220406 (PMC6660082; doi:10.1371/journal.pone.0220406)
Supplement: S2 Table — (PDF) [file pone.0220406.s007.pdf]

Table S2. Gender, age, and transaminase (ALT and AST) levels for each serum sample donated per healthy volunteer (N=240).

ALT and AST Values Used in Establishment of Reference Intervals														
Donor Information			ALT (U/L)						AST (U/L)					
Donor Number	Gender	Age	T0	T1	T2	T3	T4	T5	T0	T1	T2	T3	T4	T5
V1	M	54	32	34	38	33	28	28	25	26	28	24	22	22
V2	F	34	16	11	13	12	14	10	16	18	16	14	14	15
V3	F	55	27	27	27	30	38	49	19	20	20	22	27	32
V4	M	45	22	29	22	19	27	19	19	30	23	21	34	25
V5	F	45	13	11	13	12	15	12	16	14	16	14	16	14
V6	M	41	15	16	17	15	14	14	20	20	22	20	19	22
V7	F	43	19	12	15	12	14	11	20	17	21	18	19	16
V9	M	46	14	15	17	19	15	16	14	14	16	16	13	16
V10	F	31	11	11	8	9	9	9	16	14	13	14	13	14
V11	M	46	22	22	23	27	22	22	18	19	20	21	18	18
V12	M	48	52	37	26	37	30	26	27	20	18	19	19	14
V13	M	38	13	15	15	15	14	14	13	13	13	18	15	11
V14	F	37	21	20	17	20	22	20	24	22	20	23	28	20
V15	M	44	21	21	20	20	22	23	19	19	19	18	21	21
V16	M	41	29	29	31	27	26	28	20	21	20	17	17	18
V17	M	33	17	22	17	21	19	18	15	28	17	17	16	18
V18	M	35	13	15	13	11	11	13	16	17	15	15	15	14
V19	F	45	23	20	16	9	12	11	21	15	19	12	12	12
V20	F	58	18	18	20	17	18	19	16	16	17	15	15	15
V21	M	48	25	26	26	29	22	29	20	20	16	22	18	21
V22	M	41	14	12	12	20	41	13	13	10	14	27	63	11
V23	F	34	14	19	16	21	15	15	20	22	18	29	20	17
V24	F	41	12	11	14	10	9	9	17	17	20	16	14	16
V25	M	52	33	23	26	29	25	28	23	17	19	21	19	17
V26	F	44	16	14	13	15	14	14	16	17	15	19	18	16
V27	M	43	17	16	15	18	16	17	16	15	13	17	16	15
V28	F	35	14	20	14	16	19	17	15	19	17	19	20	17
V29	F	40	13	12	10	14	13	13	15	14	14	14	14	15
V30	F	33	15	16	14	21	15	14	19	16	16	18	17	19
V31	M	36	14	15	15	13	13	14	21	18	20	15	17	21
V32	F	46	12	12	11	12	11	12	16	20	15	17	16	17
V33	F	51	17	15	18	21	16	17	23	21	25	27	20	22
V34	M	53	23	15	19	18	31	17	22	17	21	21	25	19
V35	M	27	23	22	35	60	29	30	17	20	34	46	20	28
V36	M	32	42	41	41	42	41	47	25	27	25	26	25	27
V37	M	30	15	15	15	19	16	18	18	17	19	22	21	29
V38	M	30	22	20	20	22	22	21	23	22	23	22	22	27
V39	M	63	16	19	15	19	13	15	16	18	15	19	17	17
V40	M	40	23	21	22	20	32	21	20	21	19	26	26	20
V41	F	31	8	8	8	7	9	7	14	14	13	13	12	13
