# Supplementary material for: Variance component analysis of circulating miR-122 in serum from healthy human volunteers
Source: PLoS One. 2019 Jul 26;14(7):e0220406. doi: 10.1371/journal.pone.0220406 (PMC6660082; doi:10.1371/journal.pone.0220406)
Supplement: S3 Table — (PDF) [file pone.0220406.s008.pdf]

Table S3: Panel of miRNAs included in our exploratory panel, with percentage of useable/detectable data across all samples tested.

miRNA name	Completion	miRNA name	Completion	miRNA name	Completion
C eleg miR-39	>95%	miR-335-5p	94%-51%	miR-187-3p	<1%
miR-122	>95%	miR-133a	94%-51%	miR-222-3p	<1%
miR-let7b	>95%	miR-103-3p	94%-51%	miR-374b*	<1%
miR-17-5p	>95%	miR-210	94%-51%	miR-378a-5p	<1%
miR-19b-3p	>95%	miR-27a-3p	94%-51%	miR-106b	<1%
miR-20a-5p	>95%	miR-18a-5p	94%-51%	miR-147	<1%
miR-20b	>95%	miR-192	94%-51%	miR-147b	<1%
miR-106a	>95%	miR-511-5p	94%-51%	miR-155	<1%
miR-let7d	>95%	miR-27a-5p	94%-51%	miR-181c	<1%
miR-10a	>95%	miR-28-3p	94%-51%	miR-187-5p	<1%
miR-125a-5p	>95%	miR-100	94%-51%	miR-200a-3p	<1%
miR-125b-5p	>95%	miR-196b	94%-51%	miR-206	<1%
miR-130a	>95%	miR-629-5p	94%-51%	miR-213	<1%
miR-139-5p	>95%	miR-378a-3p	94%-51%	miR-22-3p	<1%
miR-146a-5p	>95%	miR-99a-5p	94%-51%	miR-29b-3p	<1%
miR-146b-5p	>95%	miR-500a-5p	50%-1%	miR-30c	<1%
miR-16	>95%	miR-107	50%-1%	miR-326	<1%
miR-191	>95%	miR-181a	50%-1%	miR-342	<1%
miR-193a-5p	>95%	miR-135a	50%-1%	miR-374	<1%
miR-221-3p	>95%	miR-182	50%-1%	miR-424	<1%
miR-24	>95%	miR-223	50%-1%	miR-511-3p	<1%
miR-28-5p	>95%	miR-301a	50%-1%		
miR-29a-3p	>95%	miR-150	50%-1%		
miR-301	>95%	miR-9	50%-1%		
miR-339-3p	>95%				
miR-339-5p	>95%				
miR-375	>95%				
miR-451	>95%				
miR-484	>95%				
miR-92a	>95%				
miR-143	>95%				
miR-21	>95%				
miR-23a-3p	>95%				
miR-483-5p	>95%				
miR-145	>95%				
miR-26b	>95%				
miR-29c-3p	>95%				
