# Supplementary material for: Variance component analysis of circulating miR-122 in serum from healthy human volunteers
Source: PLoS One. 2019 Jul 26;14(7):e0220406. doi: 10.1371/journal.pone.0220406 (PMC6660082; doi:10.1371/journal.pone.0220406)
Supplement: S4 Table — (PDF) [file pone.0220406.s009.pdf]

Table S4. Reference interval (95% CI) reported as fold-change of expression for miR-122 expression in serum from volunteers that identified as Caucasian, Non-Caucasian, and total volunteer cohort when expression was normalized using miRA-norm or to *C eleg* miR-39.

Gene	Population	miRA-norm	*C. eleg* miR-39
cel-miR-39	Non-Caucasian	7	NA
	Caucasian	8	NA
	Total	7	NA
miR-122	Non-Caucasian	241	239
	Caucasian	66	62
	Total	117	111
miR-let7b	Non-Caucasian	2	6
	Caucasian	2	6
	Total	2	6
miR-17-5p	Non-Caucasian	2	8
	Caucasian	2	12
	Total	2	10
miR-19b-3p	Non-Caucasian	2	7
	Caucasian	2	7
	Total	2	7
miR-20a-5p	Non-Caucasian	2	8
	Caucasian	2	8
	Total	2	8
miR-20b	Non-Caucasian	2	9
	Caucasian	2	9
	Total	2	10
miR-133a	Non-Caucasian	61	76
	Caucasian	38	50
	Total	45	61
miR-let7d	Non-Caucasian	8	7
	Caucasian	8	9
	Total	7	8
miR-106a	Non-Caucasian	2	7
	Caucasian	2	12
	Total	2	10
miR-125b-5p	Non-Caucasian	11	12
	Caucasian	13	12
	Total	11	12
miR-130a	Non-Caucasian	6	8
	Caucasian	6	13
	Total	6	10
miR-16	Non-Caucasian	3	8
	Caucasian	3	7
	Total	3	8
miR-24	Non-Caucasian	4	5
	Caucasian	4	6

	Total	4	5
miR-26b	Non-Caucasian	5	10
	Caucasian	7	18
	Total	6	14
miR-221-3p	Non-Caucasian	8	9
	Caucasian	6	8
	Total	7	8
miR-375	Non-Caucasian	56	57
	Caucasian	34	22
	Total	41	34
miR-483-5p	Non-Caucasian	16	16
	Caucasian	8	5
	Total	11	10
miR-92a	Non-Caucasian	4	7
	Caucasian	5	7
	Total	4	8
